# Rescue of collapsed replication forks is dependent on NSMCE2 to prevent mitotic DNA damage

**DOI:** 10.1371/journal.pgen.1007942

**Published:** 2019-02-08

**Authors:** Kelvin W. Pond, Christelle de Renty, Mary K. Yagle, Nathan A. Ellis

**Affiliations:** 1 Department of Cellular and Molecular Medicine, University of Arizona, Tucson, Arizona, United States of America; 2 University of Arizona Cancer Center, University of Arizona, Tucson, Arizona, United States of America; NIA-NIH, UNITED STATES

## Abstract

NSMCE2 is an E3 SUMO ligase and a subunit of the SMC5/6 complex that associates with the replication fork and protects against genomic instability. Here, we study the fate of collapsed replication forks generated by prolonged hydroxyurea treatment in human NSMCE2-deficient cells. Double strand breaks accumulate during rescue by converging forks in normal cells but not in NSMCE2-deficient cells. Un-rescued forks persist into mitosis, leading to increased mitotic DNA damage. Excess RAD51 accumulates and persists at collapsed forks in NSMCE2-deficient cells, possibly due to lack of BLM recruitment to stalled forks. Despite failure of BLM to accumulate at stalled forks, NSMCE2-deficient cells exhibit lower levels of hydroxyurea-induced sister chromatid exchange. In cells deficient in both NSMCE2 and BLM, hydroxyurea-induced double strand breaks and sister chromatid exchange resembled levels found in NSCME2-deficient cells. We conclude that the rescue of collapsed forks by converging forks is dependent on NSMCE2.

## Author summary

DNA damage encountered by the replication fork causes fork stalling and is a major source of mutations when not adequately repaired. Fork stalling can lead to fork collapse, that is, a state of the fork in which normal DNA synthesis cannot be resumed at the site of stalling. Collapsed forks must be rescued by replication forks initiated nearby, but little is known about the rescue mechanism by which an active fork merges with a collapsed fork. We used an inhibitor of DNA replication to generate collapsed replication forks and then studied genetic control of collapsed-fork rescue. We found that NSMCE2, which is a gene product that is known to regulate repair responses to replication stress, is required for cells to effectively rescue collapsed replication forks in order to complete DNA synthesis. DNA double strand breaks that are associated with normal collapsed-fork rescue do not accumulate in cells that are deficient for NSMCE2, suggesting that DNA breakage is part of the rescue and repair mechanism. Failure to rescue collapsed forks leads to DNA damage in mitosis and DNA damage in the following cell cycle. Our work highlights a unique role for NSMCE2 in rescue of collapsed replication forks.

## Introduction

Replication-associated DNA damage is common in human cells and can lead to the development of somatic mutations. DNA damage during replication can be induced by DNA lesion-producing chemicals, proteins bound to the DNA, DNA polymerase inhibitors, or nucleotide limitation [1]. Hydroxyurea (HU) triggers fork stalling due to nucleotide limitation through inhibition of ribonucleotide reductase [2]. Human cells exposed to HU for up to six hours are capable of restarting 80% of their replication forks [3–6]. However, forks that are stalled for 16 to 24 h are unable to restart [7], indicating they have collapsed. Collapsed replication forks must be rescued by active forks initiated at dormant origins to complete genome duplication. In both human cells and yeasts, the induction of a double strand break (DSB) is associated with repair of collapsed forks [8, 9]. Although factors essential for the formation of DSBs during fork collapse have been identified [10], the mechanism generating DSBs when the new replication forks converge with the collapsed forks is unknown.

The DNA helicase mutated in Bloom’s syndrome BLM possesses multiple functions in DNA replication fork stabilization and homologous recombination (HR), which is a mechanism that operates in the repair of replication-associated DSBs [11]. Recruitment of BLM to replication forks is part of the mechanism that stabilizes forks in both unperturbed and replication-stressed cells [12, 13]. Excessive DSBs accumulate in BLM-deficient cells released from replication blockade after prolonged fork stalling [14], suggesting that BLM plays a role in collapsed-fork rescue. In the absence of BLM, after fork collapse, under-replicated DNA and unresolved HR intermediates persist into mitosis where they cause DNA damage [15, 16].

The E3 SUMO ligase NSMCE2 is a component of the SMC5/6 complex, which is present at stalled replication forks and a key component of the stalled fork proteome [17]. In budding yeast, deletion of the *NSMCE2* homolog *MMS21* is lethal; however, sumoylation-deficient hypomorphs are viable and have defects in replication-specific DNA repair [18]. These cells also accumulate excess RAD51-dependent recombination intermediates during replication stress and are deficient in HR [18, 19]. During fork stalling, MMS21 undergoes auto-sumoylation during replication stress, and it then recruits the BLM homolog Sgs1 via SUMO binding sites on Sgs1 [20, 21]. Once recruited, Sgs1 resolves HR intermediates generated during repair of damaged replication forks.

In human cells, forks adopt a RAD51-dependent structure during stalling, which resembles a Holliday junction [22]. RAD51 is required to prevent replication-induced DSBs, and RAD51 levels increase at stalled forks as they transition from a restart-competent state to a collapsed state [7]. BLM regulates the exchange of RAD51 recombinase for RPA [23, 24], and in previous work we showed that sumoylation of BLM regulates a switch between BLM’s pro- and anti-recombinogenic functions [14]. If negative regulators of RAD51 such as BLM and the recently described RADX are ablated, excess RAD51 is loaded at stalled forks and excess DSBs accumulate [25]. In other situations, however, induction of excessive RAD51 can instead trigger inhibition of HR repair [26]. Because NSMCE2 regulates BLM recruitment and RAD51-dependent HR intermediates accumulate in yeast *mms21* mutant cells, we hypothesized that NSMCE2 may be a critical regulator of RAD51 function at collapsed replication forks.

Here we studied the role of NSMCE2 in repair and rescue of collapsed replication forks. We found that NSMCE2 is essential for formation of DSBs during collapsed-fork rescue. Interestingly, lack of DSBs during collapsed-fork rescue is associated with hyper-accumulation of RAD51 and impaired sister chromatid recombination. Defects in the rescue of collapsed replication forks in NSMCE2-deficient cells lead to DNA damage in mitosis.

## Results

We used two different siRNAs to deplete NSMCE2 in HeLa cells, resulting in an approximately 80% reduction in both RNA and protein levels (S1A Fig). To corroborate key NSMCE2-deficient phenotypes, we also prepared *NSMCE2-/-* clones of HEK293T cells in which we targeted a single site in exon 2 of *NSMCE2* and isolated two clones with different frameshift mutations containing no detectable NSMCE2 protein (S1B Fig). Hereafter, we refer to cells in which we have knocked down NSMCE2 as NSMCE2-deficient cells and we refer to *NSCME2-/-* cells as NSMCE2 null cells.

### NSMCE2 is required for BLM sumoylation and its localization to stalled replication forks

**Fig 1:**
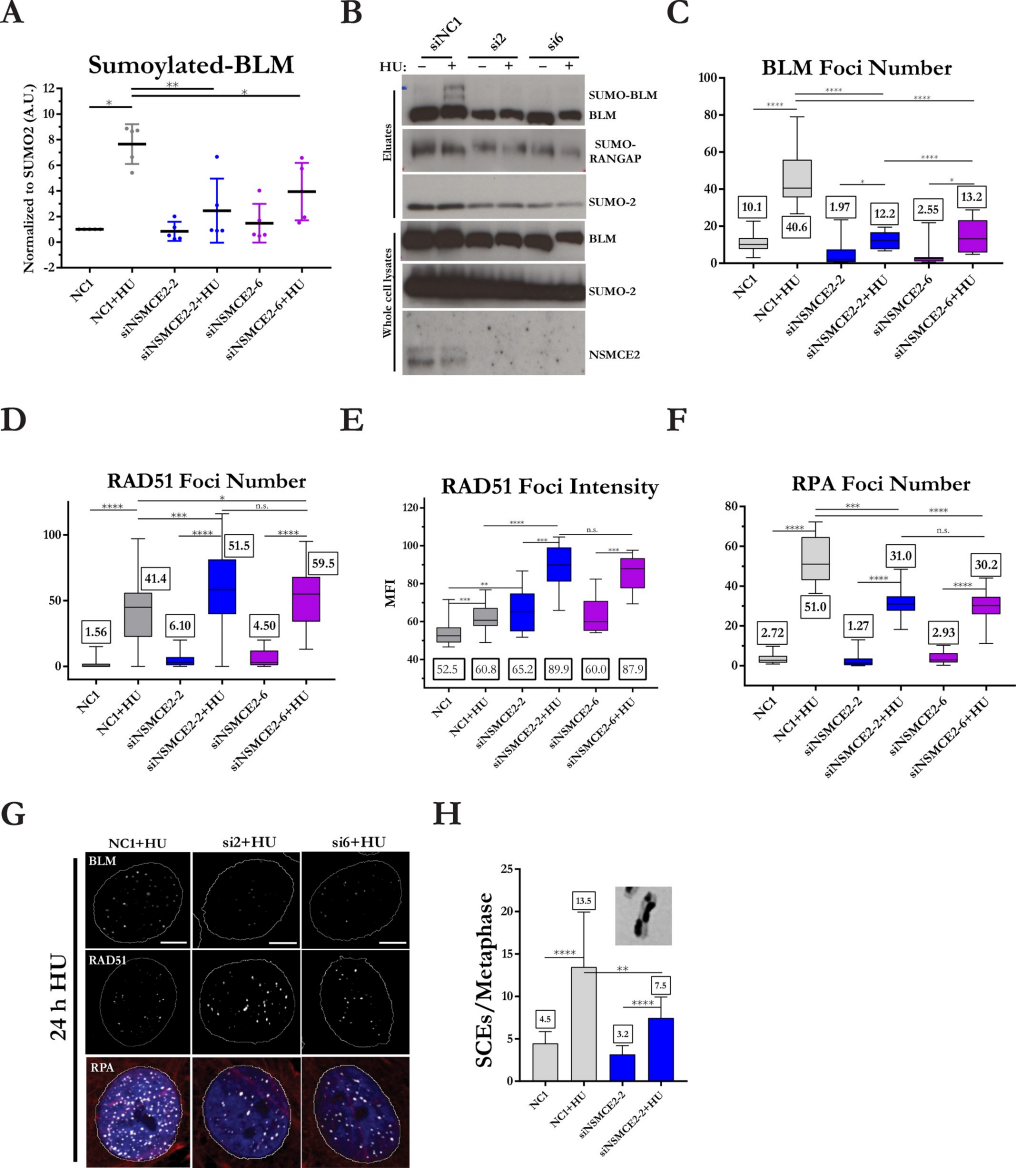
NSMCE2 is required for normal accumulations of BLM, RAD51, and RPA at stalled replication forks. (A) Depletion of NSMCE2 is associated with a reduction in SUMO2-BLM. Quantitation of SUMO2-BLM in U2OS cells normalized to controls (NC1). SUMO2-conjugates were pulled down from U2OS cells that stably overexpress His-tagged SUMO2 protein. NSMCE2 was depleted with two different siRNAs, and cells were treated or not with 2 mM HU for 16 hours. The levels of SUMO2-BLM pulled down were normalized to the amounts of SUMO2. Shown are the mean and standard deviation of five independent experiments. (B) Representative images of western blots quantitated in (A). (C) Quantitative analysis of the number of BLM foci in HeLa cells. NSMCE2 was depleted in HeLa cells with two different siRNAs, and cells were treated or not with 2 mM HU for 24 hours. Box and whisker plots represent cell distributions of BLM foci per cell. Numerical labels represent the median value of at least 10,000 BLM foci in each experimental condition. Three independent experiments were performed. (D) Quantitative analysis of the number of RAD51 foci per cell. Plots and analysis performed as in panel C. RAD51 and RPA staining were performed in the same experiment. Three independent experiments were performed. (E) Quantitative analysis of the median fluorescence intensity of RAD51 per cell. (F) Quantitative analysis of the number of RPA foci per cell. (G) Representative immunofluorescence images of RAD51 and RPA foci in cells analyzed in (D-F), showing colocalization of RAD51 and RPA. Scale bars represent 10 microns. (H) SCEs in HeLa cells exposed to control or NSMCE2 siRNA and treated or not with 2 mM HU for 24 hours. Mean and standard deviation of the number of SCEs/metaphase is shown. Three independent experiments were performed. Asterisks represent statistical analysis by paired t-test (A,H) or Mann-Whitney test (C-F) (* = p<0.05, ** = p<0.01, *** = p<0.001, **** = p<0.0001).

Experiments in yeast suggested that NSMCE2 is required for efficient sumoylation of BLM [20]. To measure SUMO-BLM levels, we used human U2OS cells that express a His-tagged SUMO2 to carry out pull down assays. Analysis of SUMO-conjugates revealed that sumoylated BLM levels increased approximately eight fold after prolonged fork stalling by treatment with 2 mM HU for 16 hours (Fig 1A and 1B). We then tested if sumoylation of BLM is dependent on NSMCE2 by knockdown of endogenous NSMCE2. Depletion of NSMCE2 using two different siRNAs resulted in a 60% decrease in sumoylated BLM in HU-treated cells compared to HU-treated control cells (Fig 1A and 1B). These data indicate that BLM sumoylation is dependent on NSMCE2. Residual SUMO-BLM could result from the incomplete depletion of NSMCE2 or from residual sumoylation catalyzed by other E3 SUMO ligases.

We previously reported that a BLM protein mutated at its preferred sumoylation sites K317R and K331R is recruited normally to stalled replication forks [14]; consequently, we hypothesized that BLM localization at stalled forks might be normal in NSMCE2-deficient cells. On the contrary, siRNA-mediated depletion in HeLa cells led to three-fold reduction in BLM foci in cells treated with HU for 24 h compared to HU-treated control cells (Fig 1C). NSMCE2 depletion was not associated with a change in the levels of BLM protein in NSMCE2-deficient HeLa cells (S2A Fig), and overexpression of siRNA-resistant *NSMCE2* in depleted HeLa cells substantially rescued the defect in localization of BLM at stalled forks (S3A Fig; representative low power images of BLM localization are shown in S4A Fig). These data show that efficient recruitment to or retention of BLM at collapsed replication forks is dependent on NSMCE2.

### NSMCE2 prevents excessive accumulation of RAD51 and promotes HR

BLM can promote dissociation of RAD51 recombinase from ssDNA [23]. Because BLM’s role in dissociation of RAD51 at stalled forks could be defective in NSMCE2-deficient cells, we tested whether NSMCE2 plays a role in regulation of RAD51 accumulation. NSMCE2-deficient cells, both untreated cells and cells treated with HU for 24 hours, exhibited increases in the number, intensity, and size of RAD51 foci compared to controls (Fig 1D and 1E; S3B Fig; representative low power images of RAD51 localization are shown in S4C Fig). Western blot analysis showed that total cellular RAD51 protein levels were similar in NSMCE2-deficient and control cells (S2A Fig). Because over 90% of stalled replication forks are unable to restart after 24 hours of HU treatment [7, 27], we tested whether the excess RAD51 was localized to collapsed replication forks. To test this, we labeled nascent DNA synthesis with 10 μM EdU for 12 min prior to HU treatment, treated cells with HU for 24 hours, and then analyzed RAD51 foci. As expected, RAD51 co-localized with EdU foci in HU-treated control and NSMCE2-deficient cells (S3C Fig). These data show that NSMCE2 is required to prevent over-accumulation of RAD51 at forks under conditions that lead to their collapse.

RAD51 is normally loaded onto ssDNA by exchange with ssDNA binding protein RPA [28]. We therefore tested whether the excess RAD51 accumulation in NSMCE2-deficient cells might correlate with a diminished accumulation of RPA at stalled forks. For this experiment, we measured the accumulation of chromatin-bound RPA after nucleoplasmic extraction of cells treated with HU for 24 hours. After siRNA-mediated depletion of NSMCE2 and HU treatment, cells displayed 40% fewer RPA foci in both HeLa (Fig 1F and 1G; representative low power images of RPA localization are shown in S4B Fig) and U2OS cells (S3D Fig) compared to control cells. Overexpression of siRNA-resistant *NSMCE2* in depleted HeLa cells rescued the defect in RPA foci accumulation (S3E Fig). In addition, HU-treated HEK293T NSMCE2 null cells displayed reduced levels of chromatin-bound RPA compared to control normal cells (S3F and S3G Fig). We conclude that RAD51 accumulates in excess over RPA in NSMCE2-deficient and NSMCE2 null cells, perhaps due to a failure to recruit BLM to stalled forks.

The lower levels of RPA foci suggest that there are lower levels of ssDNA. To test this possibility, we measured the levels of ssDNA by incorporation of BrdU for two cell divisions prior to HU treatment, followed by immunodetection with anti-BrdU antibodies in non-denaturing conditions. Unlike control cells, which displayed at least a two-fold increase in BrdU foci after treatment with 2 mM HU for 24 hours, NSMCE2-deficient cells displayed no induction of BrdU foci after HU treatment (S3H Fig; representative low power images of BrdU localization are shown in S4D Fig). Thus, the lower levels of focal RPA in HU-treated NSMCE2-deficient cells are evidence of lower levels of ssDNA detectable by anti-BrdU antibodies at collapsed replication forks. Because anti-BrdU antibodies cannot detect BrdU in the ssDNA-RAD51 nucleoprotein filament [29], these results do not rule out the possibility that the excess RAD51 is bound to ssDNA.

To test whether the RAD51-bound chromatin in NSMCE2-deficient cells is competent for HR, we measured the frequency of sister chromatid exchanges (SCEs) after prolonged fork stalling by 24-hour treatment with HU. The SCE assay measures crossovers generated after resumption of DNA synthesis that can be detected in the subsequent mitosis. NSMCE2-deficient cells had a 45% reduction in the number of HU-induced SCEs/metaphase compared to control cells (Fig 1H). Thus, the excess RAD51 observed at stalled forks is not associated with increased sister chromatid recombination. Contrary to previous reports using murine cells [30], we found that basal levels of SCEs in HEK293T NSMCE2 null cells were similar to normal HEK293T cells (S3I Fig).

### NSMCE2 is required for DSBs and RAD51 resolution during collapsed-fork rescue

**Fig 2:**
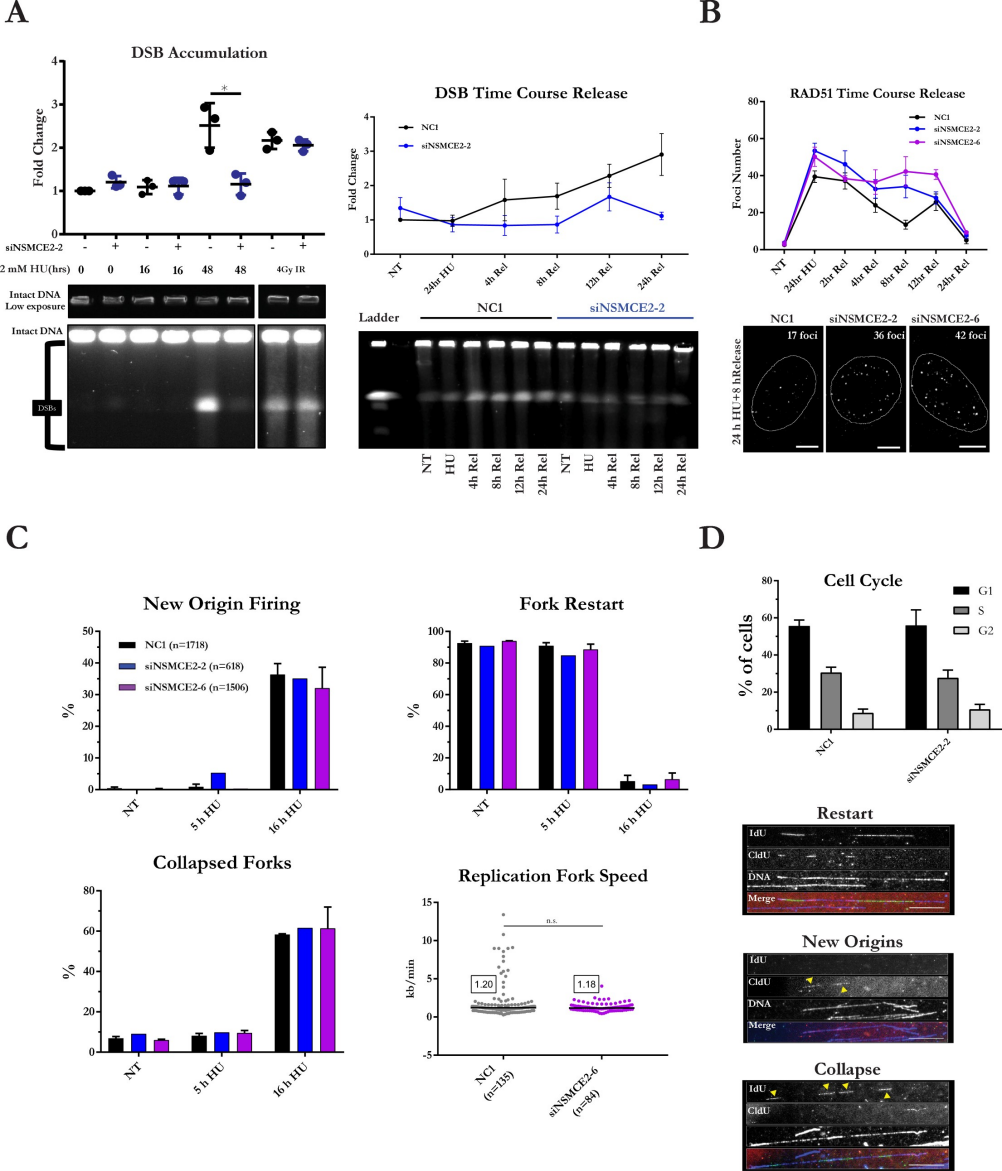
DSB formation and resolution of RAD51 foci at collapsed forks is dependent on NSMCE2. (A) Cleavage of collapsed forks is dependent on NSMCE2. Quantitative analysis (top panel on left) and representative image (bottom panel on left) of DSBs in control and NSMCE2-deficient HeLa cells treated or not with HU for 24 hours then released for 16 or 48 hours. As a control, cells were irradiated with 4 Gy and allowed to recover for 30 min. DSBs were visualized by PFGE. Three independent experiments were performed. (top panel on right) Quantitative analysis of DSBs at different times after release from HU block. Mean and standard deviation is shown. Different time courses were performed, including release times of 4 hours and 8 hours; 12 hours and 24 hours; and 4 hours, 8 hours, 12 hours, and 24 hours. In each time course, the no-treatment sample was used to normalize the data. A minimum of two independent experiments were analyzed for the 4-hour and 8-hour and the 12-hour and 24-hour time courses. (bottom panel on right) Image of a representative PFGE experiment. (B) Persistence of RAD51 foci after release from HU block in NSMCE2-deficient cells. (top panel) Quantitative analysis of number of RAD51 foci per cell at different times after release from HU block. Mean and standard error are shown. At least 10,000 RAD51 foci were analyzed in each experimental condition. Three independent experiments were performed. (bottom panel) Three representative images from the experiment. Scale bars represent 10 microns. (C) Similar replication dynamics in normal and NSMCE2-deficient cells treated with HU. DNA combing analysis by maRTA of HeLa cells exposed to control and NSMCE2 siRNAs and treated or not with 2 mM HU for 5 hours or 16 hours. Two independent experiments were performed. Four panels show quantitative analysis of new origin firing, replication fork re-start, fork collapse, and replication fork speed. The three panels below the bar graph in (D) show representative images from the maRTA. (D) Cell-cycle analysis by flow cytometry of untreated HeLa cells pulsed with 20 μM BrdU for 40 min. Mean and standard deviation is shown. Three independent experiments were performed.

Because HU-induced SCEs were suppressed in NSMCE2-deficient cells, we hypothesized that the excess RAD51 leads to a defect in DSB formation during rescue of collapsed forks. To test this possibility, we measured DSB accumulation in control and NSMCE2-deficient cells after prolonged exposure to HU. In HeLa cells exposed to control siRNA, a 16-hour treatment with HU did not induce DSBs; however, a 48-hour treatment with HU led to an accumulation of DSBs detectable by pulsed-field gel electrophoresis (PFGE) (Fig 2A). Interestingly, we found that NSMCE2-deficient cells failed to produce a detectable increase in DSBs after a 48-h exposure. Ionizing radiation with 4 Gy followed by a 30-min repair period results in equal levels of DSBs in both control and NSMCE2-deficient cells, indicating that NSMCE2-deficient cells are not defective in DSB formation per se but in replication stress-induced DSBs.

Because NSMCE2-deficient cells are defective in DSB formation at stalled forks after prolonged HU treatment, we tested whether the DNA damage response was also reduced. We measured γ-H2AX levels by analysis of DNA damage foci and flow cytometry. We found by both measures that NSMCE2-deficient cells accumulate two- to three-fold less γ-H2AX after prolonged HU treatment (S3J and S3K Fig) despite normal levels of phosphorylation of CHK1 and of RPA32 (S2A Fig). Substantial rescue of the levels of γ-H2AX foci was observed by overexpression of siRNA-resistant *NSMCE2* (S3K Fig).

To investigate the ability of cells to generate DSBs during collapsed-fork rescue, we measured the kinetics of accumulation of DSBs over time after release from HU. After release from the HU block, normal cells linearly accumulated DSBs, whereas NSMCE2-deficient cells failed to accumulate DSBs four and eight hours after release (Fig 2A). The accumulation appears to be replication-dependent, because normal cells released into 10 μM aphidicolin after HU arrest did not accumulate similar levels of DSBs (S5A Fig). Flow cytometry confirmed that control and NSMCE2-deficient cells show similar cell-cycle distributions 6 and 12 hours after release from HU, suggesting that differences in cell-cycle progression after release from HU do not explain these results (S5B Fig). Moreover, no significant differences in the levels of apoptosis were observed in control and NSMCE2-deficient cells after release from HU, ruling out apoptosis as a confounder of differences in DSBs (S5D Fig). Levels of DSBs in untreated HEK293T NSMCE2 null cells were higher than in untreated normal HEK293T cells; however, similar to the results obtained with NSMCE2 depletion with siRNAs, after treatment with HU and during release into normal medium we observed a defect in accumulation of DSBs in NSMCE2 null cells (S5C Fig). Similar to NSMCE2-deficient HeLa cells, NSMCE2 null cells were also defective in their γ-H2AX response after HU treatment (S5E Fig). Collectively, the results suggest that in the absence of NSMCE2 the levels of DSBs that are normally generated during collapsed-fork rescue are reduced.

We next tested whether NSMCE2-deficient cells have a defect in the dynamics of RAD51 localization during collapsed-fork rescue. Because RAD51 protein accumulates during HU treatment, we hypothesized that converging forks displace the RAD51 over time. We therefore released cells from prolonged fork stalling and measured levels of the RAD51 foci at collapsed replication forks in a time course. Two, four, and eight hours after release from HU, control HeLa cells exhibited a steady decrease in RAD51 foci whereas NSMCE2-deficient cells retained them (Fig 2B). RAD51 foci increased in normal cells between 8 and 12 h after release from HU, possibly due to RAD51-dependent DNA repair in late S or G2 phase. In addition, we also observed a persistence of RAD51 foci at stalled forks in NSMCE2 null cells compared to normal cells after release from HU treatment (S6A Fig). In both normal and NSMCE2 null HEK293T cells, RAD51 foci co-localized with RPA and γ-H2AX foci (S6B Fig).

We considered the possibility that persistence of excessive RAD51 at collapsed replication forks might disturb replication dynamics in S phase after release from HU. To measure replication fork dynamics, we performed microfluidics-assisted replication track analysis (maRTA)[31]. We found that replication fork speed, fork restart, and dormant origin firing were similar in NSMCE2-deficient cells compared to control cells, after either 5 or 16 hours of HU treatment (Fig 2C). These data indicate that both the replication dynamics of unperturbed forks and of dormant origin activation in replication-stressed cells are not adversely affected by NSMCE2 deficiency.

### NSMCE2 prevents mitotic DNA damage resulting from replication stress

NSMCE2-deficient cells maintained normal cell-cycle progression in the absence of HU treatment (Fig 2D); however, after release from prolonged HU treatment, NSMCE2-deficient cells displayed an arrest in the next G1 phase (S7A Fig). Defects in collapsed-fork rescue could lead to under-replicated DNA in S phase and DNA damage in mitosis. Similar to previously reported results [30, 32], we found increases in the frequencies of abnormal anaphases, micronuclei, and G1 53BP1 nuclear bodies after release from HU block in NSMCE2-deficient cells compared to controls indicating that excess mitotic DNA damage is induced in NSMCE2-deficient cells (S7B–S7F Fig; representative low power images of 53BP1 localization are shown in S4E Fig).

**Fig 3:**
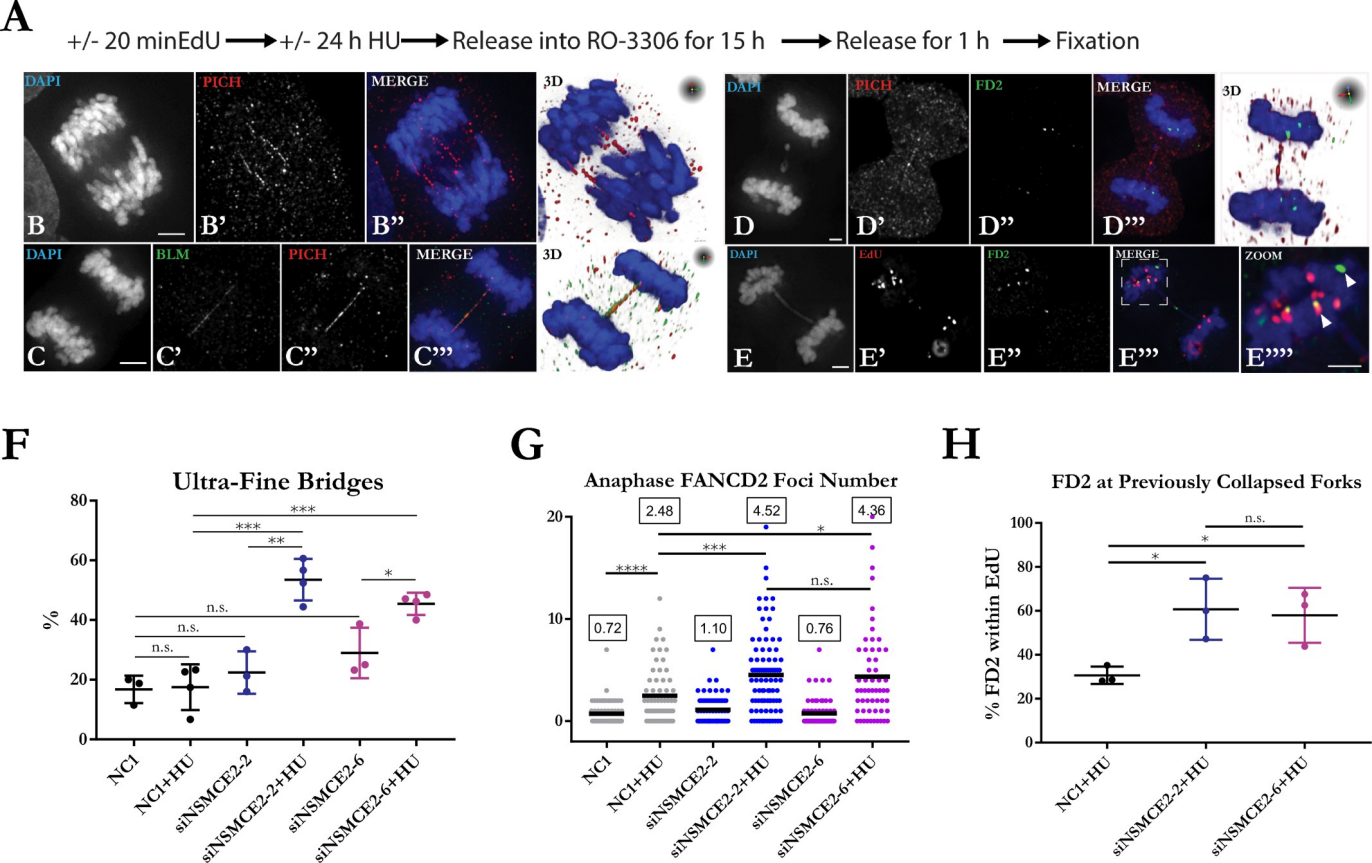
NSMCE2 prevents mitosis-dependent DNA damage. (A) Schematic for treatment and enrichment of anaphase cells for analysis of UFBs and FANCD2 foci. (B) Representative deconvoluted, maximum intensity projection images of z-stacks showing multiple PICH positive UFBs, (C) BLM/PICH colocalization, (D) FANCD2 foci flanking UFBs, and (E) EdU/FANCD2 co-localization in anaphase cells. Scale bars represent 5 microns. (F) Quantitative analysis of PICH-stained UFBs in NSMCE2-deficient and control cells, processed as in A. 30–50 anaphase z-stacks were analyzed in each experimental condition. At least three independent experiments were performed. (G) Quantitation of number of FANCD2 foci per cell in anaphase cells independent of UFBs. 30–50 anaphase z-stacks were analyzed in each experimental condition. (H) Quantitation of EdU positive anaphase cells represented as the percent of FANCD2 foci localized in regions with EdU signal. At least 100 FANCD2 foci were analyzed in each experiment in cells positive for EdU signal only. Mean and standard deviation is shown. Three independent experiments were performed. See Fig 1 for definition of asterisks.

To investigate further the nature of the mitotic damage invoked in HU-treated, NSMCE2-deficient cells, we measured the frequency of ultra-fine bridge (UFB) formation in cells undergoing mitosis. In order to obtain a sufficient number of cells in anaphase, cells were pretreated or not with HU for 24 hours; they were then blocked in G2 with the CDK1 inhibitor RO-3306 at 7.5 μM for 15 hours and then finally released into metaphase for 1 hour prior to fixation (Fig 3A). Flow cytometry confirmed effective G2 arrest by RO-3306 treatment (S7G Fig). We visualized UFBs using the PICH repair helicase, which localizes to UFBs and DNA under tension [3]. The number of UFBs measured by PICH staining was increased after HU treatment in NSMCE2-deficient cells but not control cells (Fig 3B–3D and 3F). PICH-positive UFBs were also positive for BLM (Fig 3C), indicating that localization of BLM to these structures is not dependent on NSMCE2. The crosslink repair protein FANCD2 is sometimes associated with the ends of UFBs, and has been used as a marker for under-replicated DNA persisting into mitosis [3,16]. FANCD2-flanked, PICH-positive UFBs (Fig 3D) were infrequently observed in both NSMCE2-deficient and control cells. Thus, the excess UFBs produced in NSMCE2-deficient cells are not equivalent to the UFBs produced in cells treated with low-dose aphidicolin [15, 16].

We then tested whether mitotic DNA damage originated from collapsed forks in the previous S phase. As a marker for damaged DNA and repair in anaphase cells, we counted the number of FANCD2 foci and found a 1.8 fold increase in NSMCE2-deficient cells treated with HU compared to control cells (Fig 3E and 3G). In order to monitor the location of collapsed forks generated by prolonged treatment with HU, we labeled cells with EdU for 20 min before treatment with HU. FANCD2 foci co-localized with the EdU label at a greater frequency in HU-treated, NSMCE2-deficient cells compared to control cells (Fig 3H), indicating that the recruitment of FANCD2 observed in mitosis had occurred in regions of chromatin where replication forks had previously stalled and collapsed. These data suggest that defective collapsed-fork rescue in NSMCE2-deficient cells leads to increased under-replicated DNA persisting into mitosis, which results in mitotic DNA damage.

In order to measure DNA damage in metaphase cells, we measured the levels of γ-H2AX associated with metaphase chromosomes and the levels of chromosome aberrations detectable at metaphase. For the analysis of γ-H2AX levels, normal HEK293T and NSMCE2 null cells were treated with 2 mM HU for 24 hours, released into medium with RO-3306 to block them in G2, then released into normal medium and prepared for analysis of γ-H2AX levels by immunofluorescence (S8A and S8B Fig). HU-treated NSMCE2 null cells exhibited a nearly 50% increase in median fluorescence intensity of chromosome-associated γ-H2AX in phospho-H3-positive cells compared to HU-treated HEK293T normal cells. For the analysis of chromosome aberrations, cells were treated or not with 2 mM HU for 24 hours and then metaphase chromosomes were prepared and analyzed by fluorescence microscopy (S9A Fig). We identified increased frequencies of chromatid arm breaks, telomere fusions, and secondary constrictions in NSMCE2 null cells compared to control cells. Chromatid arm breaks and secondary constrictions were induced by HU treatment (S9B Fig). The increase in secondary constrictions in HU-treated NSMCE2 nulls cells is consistent with increased under-replicated DNA and the increase in chromatid breaks could arise from chromosome breakage in mitosis or under-replication as seen at common fragile sites.

### Epistasis relationships among *NSMCE2*, *BLM*, and *RAD51* during collapsed-fork rescue

**Fig 4:**
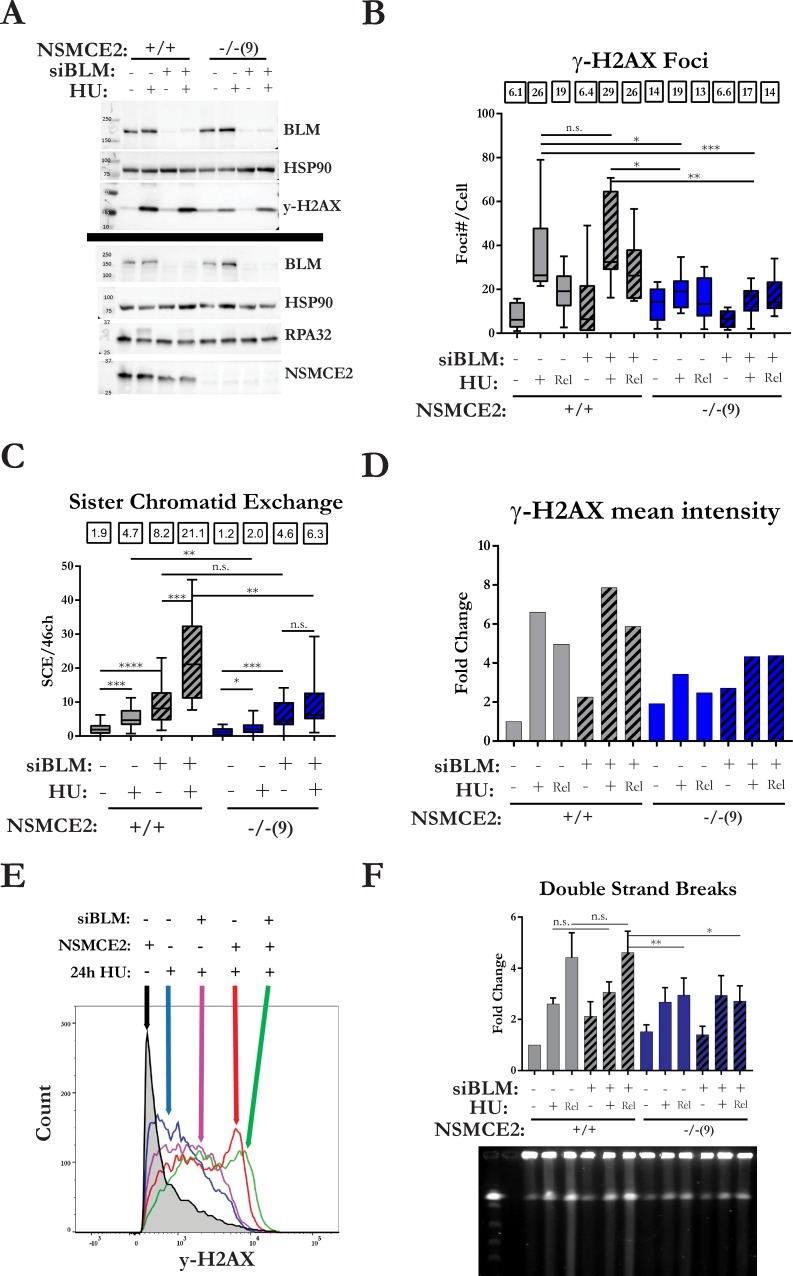
*NSMCE2* is epistatic to *BLM* for HU-induced phenotypes. Analysis of HU-induced phenotypes in normal HEK293T and NSMCE2 null cells in which BLM levels were reduced or not by siRNA-mediated depletion. (A) Western blot analysis of levels of phosphorylation of H2AX and RPA. Black line indicates separate blots using lysates from the same experiment. The experiment was performed two times. (B) Analysis of focal concentrations of γ-H2AX after treatment with HU for 24 hours and after release into normal medium for 6 hours. Box and whisker plots representing at least 10,000 γH2AX foci in two independent experiments. Medians are shown above the graph. (C) Levels of HU-induced SCEs. Box and whisker plots representing distribution of SCE per metaphase. Medians of the combined data are shown above the graph. Two independent experiments were performed. Unpaired t-test was performed to analyze the distributions. (D) Analysis by flow cytometry of fluorescence intensity of γ-H2AX after treatment with HU for 24 hours and after release into normal medium for 6 hours. Median florescence intensity was measured from a minimum of 10,000 events. The bar graph represents the fold change in median fluorescent intensity normalized to untreated normal HEK293T cells exposed to control siRNA from a single experiment. Two independent experiments were performed. (E) Representative distributions of fluorescence intensity of cells in selected conditions from the flow cytometry data. (F) Analysis by PFGE of DSBs after treatment with HU for 24 hours and after release into medium for 6 hours. The bar graph represents fold change in DSBs detected by PFGE normalized to untreated normal NSMCE2 cells exposed to control siRNA. The error bars represent the SEM of three independent experiments. The gel below the bar graph contains the results from one experiment. See Fig 1 for definition of asterisks.

Because NSMCE2 is essential for proper localization of BLM to stalled replication forks but displays phenotypes distinct from BLM-deficient cells, we asked whether *NSMCE2* is epistatic to *BLM* during rescue of collapsed forks. In these experiments, we used siRNAs to deplete BLM in NSMCE2 null and control cells (Fig 4A). In HU-treated normal HEK293T cells depleted for BLM, levels of phosphorylated RPA and γ-H2AX were similar to levels in HU-treated control cells as evidence by Western blot analysis. In contrast, in HU-treated NSMCE2 nulls cells depleted for BLM, the levels of phosphorylated RPA and γ-H2AX were reduced in comparison to HU-treated control cells, but they were similar to levels in HU-treated NSCME2 null cells. The data for phosphorylated H2AX were confirmed by analysis of focal γ-H2AX and flow cytometry with γ-H2AX antibodies (Fig 4B, 4D and 4E). Prolonged HU treatment of BLM-deficient normal HEK293T cells resulted in a 2.6 fold increase in the levels of SCEs from 8.2 to 21.1 SCEs/metaphase, whereas prolonged HU treatment of BLM-deficient NSMCE2 null cells resulted in an only small increase in SCEs from 4.6 to 6.3 SCEs/metaphase (Fig 4C). Consistent with the suppression of HU-induced SCEs, the levels of HU-induced DSBs were also suppressed in BLM-depleted NSMCE2 null cells relative to BLM-deficient normal cells. These data indicate that NSMCE2 is epistatic to BLM with respect to HU-induced phenotypes.

Because DSB accumulation is suppressed in NSMC2-deficient cells, we tested whether depletion of RAD51 would restore HU-induced DSB levels to normal in NSMCE2 null cells. After transfection of control and RAD51 siRNAs, we treated cells with 2 mM HU for 24 hours, then released into normal medium for 6 hours and quantitated the DSB marker γ-H2AX by flow cytometry and measured DSBs by PFGE. In HU-treated, control-depleted normal HEK293T cells, γ-H2AX levels increased approximately 10 fold compared to baseline after release into normal medium for 6 hours (S10A and S10C Fig). Contrary to expectation, RAD51 depletion in normal cells resulted in much smaller induction of γ-H2AX, similar to the levels observed in HU-treated control-depleted NSMCE2 null and HU-treated, RAD51-depleted NSMCE2 null cells. Consistent with the γ-H2AX results, in HU-treated, control-depleted normal HEK293T cells, DSB levels increased approximately three fold compared to baseline after release into normal medium for 6 hours (S10B Fig). In contrast, RAD51 depletion in normal cells resulted in almost no induction of DSBs compared to baseline, which again was similar to the levels of DSBs in HU-treated control-depleted NSMCE2 null and HU-treated, RAD51-depleted NSMCE2 null cells. These data suggest that, despite the fact that RAD51 has hyper-accumulated at collapsed replication forks, in the absence of NSMCE2, the RAD51 at collapsed forks is nonfunctional.

## Discussion

DNA combing experiments in many mammalian cell lines have shown that, after prolonged fork stalling due to HU exposure, DNA synthesis does not normally resume at the site where the fork stalled (see reference 2 and Fig 2). These data rule out rescue mechanisms, such as HR-mediated restart or break-induced replication, in which replication is re-established at the site of fork stalling. The majority of collapsed forks must therefore be rescued by converging forks initiated at dormant origins after release from prolonged replication arrest. DSBs have been previously associated with resumption of DNA synthesis after release from prolonged HU block [7, 14], but the mechanism by which these breaks are generated is not well understood. Here we show that normal rescue of collapsed replication forks is dependent on NSMCE2. After resumption of DNA synthesis, NSMCE2-deficient cells do not accumulate normal numbers of DSBs. The large increase in UFBs in mitosis indicates that many forks fail to complete DNA replication.

**Fig 5:**
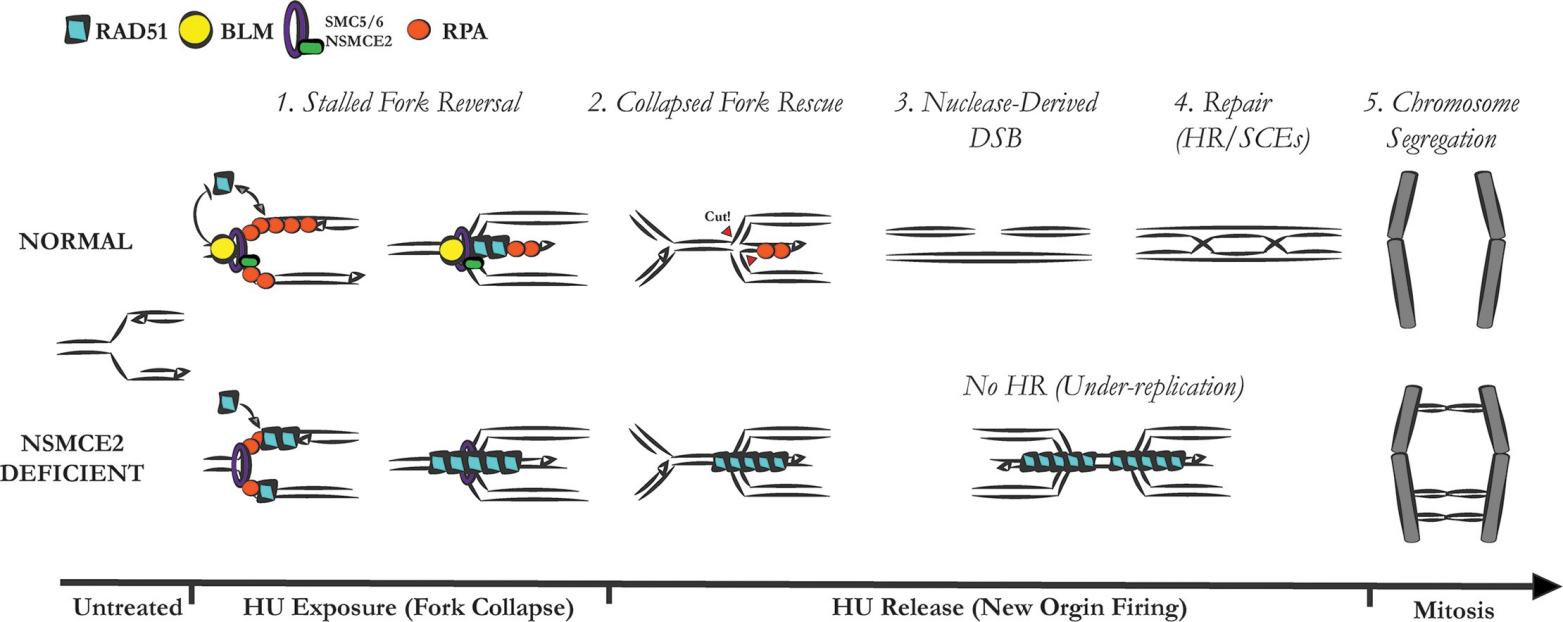
Model for collapsed-fork rescue by NSMCE2. Normally (top part of model), after exposure to HU, uncoupling of the replicative helicase from the replicative DNA polymerase at the fork leads to production of ssDNA, which is immediately bound by ssDNA binding protein RPA (red circle). BLM (yellow circle) is one of the first factors to be recruited to the stalled replication fork and this accumulation of BLM is dependent on the NSMCE2 protein (green rectangle) on the SMC5/6 complex (purple ring). The localization of BLM at the stalled fork assists in maintaining functional RAD51 (teal rhombus) exchange during fork reversal. Given enough time in the reversed configuration, the fork is unable to restart (fork collapse). After resumption of DNA synthesis at dormant origins, an active fork converges upon the collapsed fork. The convergence is associated with RAD51 dissociation and a DSB at the collapsed fork, followed by DSB repair by HR via the canonical RAD51-mediated pathway. In NSMCE2-deficient cells (bottom part of model), BLM is no longer recruited to the stalled fork, and there is a failure in the regulation of RAD51 loading at the fork, shown here as a loss of dynamic exchange of RAD51 and RPA resulting in excess, hyper-stable RAD51. After resumption of DNA synthesis at dormant origins, the aberrant RAD51 structure prevents convergence of the active fork with the collapsed fork, resulting in a double fork stall instead of a DSB. The double fork stall persists into mitosis where it results in an ultra-fine DNA bridge and mitotic DNA damage.

NSMCE2 deficiency is associated with higher levels of RAD51 foci at collapsed forks and persistence of foci after release from replication arrest. These results are consistent with results obtained in yeast mutants of the SMC5/6 complex and of *MMS21*, in which excess RAD51-dependent recombination intermediates accumulate at stalled forks [19, 33, 34]. Pathological accumulations of RAD51 have been associated with DNA repair defects [26, 35]. We do not know the structure of the RAD51-bound DNA in NSMCE2-deficient cells, and the excess RAD51 could be at the fork itself, associated with ssDNA gaps behind the fork, or associated with other abnormal structures. There are established roles for RAD51 at stalled replication forks that do not involve DSBs. For instance, RAD51’s role in reversal of the replication fork is epistatic to its BRCA2-mediated fork protection function [36, 37]. Our experiments with RAD51 depletion in normal cells suggest that reversed fork structures, catalyzed by RAD51, are required for the formation of DSBs during fork rescue, very likely as substrates for nucleases such as MUS81. Biochemical studies suggest that BLM performs a quality-control function on stressed replication forks by dissociating nonfunctional, ADP-bound RAD51 from the nucleoprotein filament [23]. Because BLM does not accumulate normally at stalled forks in the absence of NSMCE2, it is possible that the RAD51 that accumulates excessively in NSMCE2-defcient cells is in the nonfunctional ADP-bound state. That said, the epistasis experiments indicate that NSMCE2 controls other factors besides BLM that contribute to the function of RAD51 at collapsed forks. We propose a model in which the excess nonfunctional RAD51 prevents DSB formation during the convergence of active replication forks with collapsed forks, leading to excess under-replicated DNA that is detectable at anaphase (Fig 5). However, more definitive tests of this proposition are required to rule out other possible models. For example, it could be informative to use molecular combing to monitor the replication dynamics of forks from newly fired origins as they converge upon collapsed replication forks.

We found that BLM sumoylation is dependent on the presence of NSMCE2. We and others [32] have shown that BLM does not accumulate normally at stalled forks in NSMCE2-deficient cells. BLM has multiple functions in the resolution of recombination intermediates during replication stress, normally ensuring that recombination intermediates are resolved without exchange. Yet the levels of SCEs are low in the absence of NSMCE2, suggesting additional roles of NSMCE2 in promotion of crossover events when BLM is absent. Our evidence suggests that RAD51-depenedent intermediates in NSMCE2-deficient cells are not resolved until M phase, whereas RAD51 foci are normally resolved during S phase. Because BLM localization to UFBs is not dependent on NSCME2, BLM could have a role in resolving RAD51-depenedent intermediates in mitosis. Our findings that excess γ-H2AX accumulated on metaphase chromosomes but not during S phase suggest that under-replicated DNA is not resolved until G2/M phase. For example, prometaphase DNA repair [3] or mitotic resolvases [38] could decatenate under-replicated DNA to permit disjunction of inter-linked sister chromatids.

We cannot rule out the possibility that the loss of NSMCE2 in cells affects the function of the SMC5/6 complex. However, in agreement with previous results in human U2OS and DT40 cells [39, 40], we found SMC5 levels were normal in NSMCE2-deficient HeLa cells (S2B Fig). These data suggest that, unlike in *S*. *cerevisiae* [41], SMC5 levels are not dependent on the presence of NSMCE2 in human cells. Whether human NSMCE2 plays a structural role in collapsed-fork rescue and other repair processes remains to be determined.

Our results here agree with previous results showing lower levels of SCEs in NSMCE2-deficient cells [42]. Hypomorphic *NSMCE2* mutation in humans is associated with a syndrome characterized by short stature and acanthosis nigricans [32]. Cells derived from these patients display increased micronuclei, nuceloplasmic bridges at cytokinesis, and binucleated cells. Despite a defect in BLM localization at replication forks in patient cells, untreated cells have normal SCE levels, and UV treatment induces only a small increase in SCEs [32]. We found that cells deficient for both NSMCE2 and BLM exhibit reduced levels of HU-induced DSBs and SCEs, indicating that NSMCE2 is epistatic to BLM during collapsed-fork rescue. In contrast to results with human cells, murine cells that are null for *Nsmce2* exhibit increased SCEs, and *Blm* knockdowns in the murine *Nsmce2* null cells display an additive increase in SCE levels [30]. The explanation for these different outcomes of NSMCE2 deficiency between humans and mice is unknown.

We used HU to generate and study collapsed forks and to block repair-coupled DNA synthesis (e.g., break-induced replication, gap filling, lesion bypass, etc.). We therefore cannot rule out the possibility that NSMCE2 plays other roles during the unperturbed cell cycle or in situations where template switching can bypass DNA damage during replication, such as in cells treated with methyl methanesulfonate or UV irradiation. We observed higher levels of DSBs in untreated NSMCE2 null cells (S4 Fig), but observed no increase in basal SCE levels (Fig 4C). Our analysis using maRTA indicated that deficiency of NSMCE2 did not alter replication dynamics in untreated cells, which is consistent with results reported in yeast [43]. We suggest that the increased DSBs in untreated NSMCE2-deficient cells may originate from incomplete replication at common fragile sites, leading to DNA breakage in mitosis and the formation of micronuclei in the next cell cycle. Micronuclei are known to be prone to replication-associated DNA breakage [44]. HR-directed DSB repair is dependent on NSMCE2 [45, 46]; consequently, these breaks would most likely be repaired by non-homologous end joining. Studies in both mammalian cells [30, 47] and yeasts [48] indicate that HR can be increased in NSMCE2-deficient cells under some conditions, emphasizing the general complexity of NSCME2’s roles in maintenance of genomic integrity.

The importance of mechanisms that regulate RAD51 protein levels is underscored by studies that have identified increased RAD51 protein levels as a negative predictor of patient outcome in several cancer types [49]. The present work has uncovered a connection between NSMCE2 and the formation of DSBs at collapsed replication forks during rescue. The identification of NSMCE2 as a potential controller of HR-mediated fork rescue highlights NSMCE2’s potential as a new therapeutic target for combinatorial therapy of HR-dependent cancers.

## Materials and methods

### Antibodies and siRNAs

Antibodies for immunofluorescence and western blots were obtained as follows: anti-RPA2 (Abcam; mouse monoclonal ab2175), anti-RAD51 (Abcam; rabbit monoclonal ab133534), anti-NSMCE2 (OriGene; mouse monoclonal TA501632), anti-BLM [50], anti-RANGAP (Thermo Fisher Scientific; rabbit polyclonal PA1-5866), anti-histone H3 (Cell Signaling Technology; rabbit polyclonal 9715), anti-PCNA (OriGene; mouse monoclonal TA800875), anti-SMC5 (Bethyl; rabbit polyclonal A300-236A), anti-HSP90 (OriGene; mouse monoclonal TA500494), γ-H2AX (BioLegend; 613406; or Upstate; mouse monoclonal 05–636), anti-bromodeoxyuridine (BrdU) (Bio-Rad; mouse monoclonal OBT0030), anti-RPA p70 (Santa Cruz; mouse monoclonal SC-53497), anti-PML (Santa Cruz; mouse monoclonal SC-966), Phalloidin-546 (Thermo Fisher Scientific; Alexa Fluor A22283), and anti-phospho-histone H3 (serine 10; Cell Signaling; mouse monoclonal 9706). siRNAs for NSMCE2, BLM, and RAD51 were as follows:

siNSMCE2-2: 5'-rUrUrArCrArUrArArUrGrGrUrUrUrArGrUrUrGrCrCrGrArUrCrCrA-3'

5'-rGrArUrCrGrGrCrArArCrUrArArArCrCrArUrUrArUrGrUdAdA-3'

siNSMCE2-6: 5'-rUrArUrArUrUrCrArCrUrArCrUrCrArCrUrUrCrArGrUrCrUrGrArC-3'

5'-rCrArGrArCrUrGrArArGrUrGrArGrUrArGrUrGrArArUrAdTdA-3'

siBLM: 5’-rArUrUrCrUrUrGrArGrArGrCrArGrUrArUrCrCrCrGrGrGrArUrU-3’

5’-rUrCrCrCrGrGrGrArUrArCrUrGrCrUrCrUrCrArArGrAdAdAdT3’

siRAD51: 5’-rGrArGrCrUrUrGrArCrArArArCrUrArCrUrU-3’

5’-rCrArCrCrUrUrGrArArGrUrArGrUrUrUrGrU-3’

### Genome editing for NSMCE2

Genome editing was carried out with Integrated DNA Technologies (IDT) ALT-R CRISPR-Cas9 system with crRNA guide (GTCCATACCAGAGTTGATAC) targeting the first coding exon. Ribonucleoprotein particles were introduced into HEK293T cells using FuGENE HD (Promega). After 48 hours, single cells were deposited in a 96-well plate by flow cytometry. After four to six weeks, clones were analyzed by the T7 endonuclease assay (New England Biolabs), and clones that scored positively were PCR sequenced. >50 clones were analyzed in the first screen and a single heterozygous NSMCE2 null mutant was obtained. This clone was genome edited again to obtain two NSMCE2 null mutants.

### Western blot analysis

Cells were lysed in RIPA buffer (150 mM NaCl, 1% Triton X-100, 0.25% sodium deoxycholate, and 50 mM Tris-HCl, pH 8.0) supplemented with 5 mM EDTA, 1 mM EGTA, 25 mM sodium fluoride, 1 mM sodium orthovanadate, 1 mM phenylmethane sulfonyl fluoride (PMSF) in 1x EDTA-free Halt protease inhibitor (Thermo Scientific). Protein concentration was measured using Pierce BCA Protein Assay. 30–50 μg of total protein from cell lysates was separated by electrophoresis through 4–20% gradient polyacrylamide gels and transferred onto Hybond nitrocellulose membranes by semi-dry transfer. Before addition of primary antibodies, membranes were probed with Ponceau S (Sigma) for 7 min, imaged, and washed with 1% glacial acetic acid in water. Membranes were blocked for 1 hour in Tris-buffered saline with 0.1% polysorbate 20 (TBST) containing 5% Bio-Rad Blotting-Grade Blocker, then incubated with primary antibody in 3% BSA in TBST overnight at 4°C.

### SUMO2 pull-down

U2OS cells that were stably transfected with a His-tagged SUMO2 were kindly provided by Dr. Michael Matunis at Johns Hopkins University, who obtained them from Dr. Mary Dasso’s lab at NIH. The cells were reverse-transfected with siRNAs using LifeTechnologies’ Lipofectamine RNAiMAX. Cells were treated with 2 mM HU for 16 hours. His-SUMO2 conjugates were purified as described by Tatham et al. [51]. Briefly, cells were washed with ice-cold phosphate-buffered saline (PBS) and directly lysed in 4 ml lysis buffer (6 M guanidine-HCl, 100 mM NaCl, 10 mM Tris-HCl pH 7.4, 3 mM imidazole and 2 mM β-mercaptoethanol) and sonicated to reduce viscosity. Lysates were incubated with 50 ml Talon Metal Affinity Resin (Clontech Laboratories, Inc.) overnight at 4°C with gentle mixing, and then washed 2 times with 4 ml guanidine wash buffer (6 M guanidine-HCl, 100 mM NaCl, 10 mM Tris-HCl pH 7.4, 0.1% Triton X-100, 2 mM β-mercaptoethanol) followed by 3 washes in 4 ml urea wash buffer (8 M urea, 100 mM NaCl, 10 mM Tris-HCl pH 7.4, 0.1% Triton X-100, 2 mM β-mercaptoethanol). The beads were transferred to 1.5 ml microfuge tubes for one more wash with urea wash buffer and proteins were eluted for 1 hour at room temperature with elution buffer (63 mM Tris-HCl pH 6.8, 2% SDS, 200 mM imidazole, 1.5% β-mercaptoethanol, 10% glycerol, with bromophenol blue). The eluates were boiled for 5 min and cleared by centrifugation prior to loading on a 10% polyacrylamide gel. The whole cell lysates were lysed in RIPA buffer. Laemmli buffer was added to 1X in aliquots representing 10% of the eluate, then boiled for 5 min prior to gel loading.

### Analysis of chromatin-bound RPA

The protocol from Mendez and Stillman for chromatin isolation by small-scale fractionation was followed [52]. HEK293T normal and NSMCE2 null cells treated or not with 2 mM HU for 16 hours were harvested by scraping, centrifuging, and washing twice with PBS. The cells were resuspended such that there were 1 x 10^7^ cells per 200 μl of Buffer A (10 mM HEPES pH 7.9, 10 mM KCl, 1.5 mM MgCl_2_, 0.34 M sucrose, 10% glycerol, 1 mM DTT, 0.1 mM PMSF, and 1x Protease Inhibitor Cocktail). Triton X-100 was added to a concentration of 0.05%, and the cells were incubated for 5 min on ice. Nuclei were collected by low-speed centrifugation (4 min, 1300 x g at 4°C) and the supernatant was reserved as the cytoplasmic fraction. The nuclei were washed once in Buffer A, and then lysed in 100 μl Buffer B (3 mM EDTA, 0.2 mM EGTA, 1 mM DTT, 1x Protease Inhibitor Cocktail). Nucleoplasmic proteins were separated from chromatin-bound proteins by centrifugation (5 min, 1700 x g at 4 ^o^C). Nucleoplasmic fractions were collected in the supernatant. The chromatin pellet was resuspended in 250 μl Laemmli buffer and the material was sonicated. The cytoplasmic and nucleoplasmic fractions were clarified by high-speed centrifugation (5 min, 20,000 x g at 4°C). The proteins in the fractions were analyzed by Western blot. Initially, cytoplasmic and nucleoplasmic fractions were analyzed separately; however, because the cytoplasmic fraction contained varying amounts of different RPA components, for comparisons of the amounts of RPA70 we combined equal parts of the cytoplasmic and nucleoplasmic fractions.

### Immunofluorescence and image analysis

The protocol was adapted from Dimitrova and Gilbert [53]. Cells were grown on coverslips overnight and washed with cold CSK buffer (10 mM HEPES pH 7.4, 300 mM sucrose, 100 mM NaCl, 3 mM MgCl_2_) and nucleoplasm was extracted for 90 seconds with cold extraction buffer (0.5% Triton X-100 in CSK buffer with 1 mM PMSF, 50 mM sodium fluoride, 0.1 mM sodium orthovanadate and 1x EDTA free Halt protease inhibitor) prior to 30-min fixation in 4% formaldehyde at room temperature. Cells were washed twice with cold PBS then treated with 0.5% Triton X-100 at room temperature before staining. Cells were blocked at room temperature for 1 hour using sterile filtered 3% BSA in PBS, then probed using primary antibodies for 1 hour at room temperature. Secondary antibodies (Alexa Fluor 488 and 546) were used at 1:1000 for 45 min and nuclei were stained using Molecular Probes NucBlue reagent (R37606). For 5-ethynyl-2´-deoxyuridine (EdU) labeling, cells were incubated in 10 μM EdU for 20 min. EdU labeling and detection was performed using Life technologies Click-iT EdU Alexa Fluor 647 imaging kit according to manufacturer’s instructions. Cells were mounted in Molecular Probes ProLong Gold Antifade Reagent. Fixed and stained cells were imaged using the Leica SP5-II spectral confocal microscope using the 63x/1.4 NA PL Apo objective. Using the nuclear signal to mask the region of interest enabled accurate measurement of the number and intensity of nuclear foci and the percent of nucleus occupied by signal for each antibody target. No less than 10,000 foci were analyzed per experimental group. Box and whisker plots were used to visualize the distribution of foci. For analysis of EdU labeled forks and ultra-fine bridges (UFBs), z-stacks were created using 100x objective and deconvolved using the GE DeltaVision Elite High Resolution Microscope. Images were analyzed and 3D representations were created using the NIS Elements software.

### Flow cytometry

Cells were harvested with trypsin/EDTA, resuspended in ice cold PBS, and fixed and stained using BioLegend True-Nuclear Transcription Factor Buffer. Cells were then stained for γ-H2AX using directly conjugated antibody and counterstained using 7AAD to monitor cell DNA content. For cell-cycle assays, analysis was performed using the BD Pharmingen FITC BrdU Flow Kit. A minimum of 30,000 events was recorded for each group using the BD FACSCanto II or BD LSR II flow cytometer. Apoptosis analysis was carried out using the BioLegend FITC Annexin V Apoptosis Detection Kit with propidium iodide (PI).

### Cytogenetic analysis

For SCE analyses, cells were cultured with 10 μM BrdU (Sigma-Aldrich). After 60 hours, the cells were incubated with 0.02 μg per ml colcemid (Invitrogen) for up to 2 hours, harvested and processed as described earlier [14]. For the epistasis experiments using HEK293T cells, the cells were incubated in 0.6 μg per ml colcemid for 16 hours prior to harvest. The slides were examined under the microscope at 100×, and SCEs were counted from metaphases with an acceptable quality of sister-chromatid discrimination. For measurements of HU-induced SCEs, cells were cultured in 10 μM BrdU for 30 hours, washed one time with 1× PBS, and treated with 2 mM HU for 24 hours. Cells were then released into medium containing 10 μM BrdU for an additional 20 hours. Metaphases were collected in colcemid and processed as described above.

For analysis of micronuclei, normal or NSCME2-depleted HeLa cells were seeded onto coverslips, treated or not with 2 mM HU for 24 hours, and fixed with 4% formaldehyde. The cells were stained with Nuc-Blue (Thermo Fisher) at 2 drops per ml in PBS for 30 min, washed briefly, mounted, and imaged using the GE DeltaVision Elite High Resolution Micro.

For analysis of chromosomes at metaphase, normal and NSMCE2 null HEK293T cells were seeded into 60 mm dishes and incubated overnight. For analysis of γ-H2AX labeled chromosomes, cells were treated with 2 mM HU for 24 hours, then washed and incubated in medium with 7.5 μM RO-3306 for 10 hours for HEK293T control or 20 hours for NSMCE2 null cells. Cells were then released into normal medium and harvested at the indicated time points. Cells were fixed with 4% formaldehyde and stained with anti-phospho-histone H3 (serine 10). For analysis of chromosome spreads, cells were treated with 0.02 μg per ml colcemid for 45 min or with 0.6 μg per ml colcemid for 16 hours prior to harvest. Cells from both experiments were harvested and metaphases prepared as described [14]. Metaphase chromosomes were stained with Nuc-Blue. Cells or chromosomes were imaged using the GE DeltaVision Elite High-Resolution Microscope.

### DSB detection by pulsed-field gel electrophoresis

The procedure was performed as previously described [8, 14]. Sub-confluent cultures of HeLa cells or HEK293T cells untreated, treated with 2 mM HU for 24 hours, or treated with HU for 24 hours and released into normal medium for different times were harvested by trypsinization. Agarose plugs of 2.5 x 10^5^ cells were prepared in disposable plug molds (Bio-Rad Laboratories). In some experiments, cells were released into medium containing 10 μM aphidicolin for 24 hours. Plugs were then incubated in lysis buffer (100 mM EDTA, 1% wt/vol sodium lauroyl sarcosinate, 0.2% wt/vol sodium deoxycholate, and 1 mg/ml proteinase K) at 37°C for 16 hours. Plugs were then washed four times in 20 mM Tris-HCl, pH 8.0, and 50 mM EDTA before loading onto an agarose gel. Electrophoresis was performed for 21 hours at 14°C in 0.9% (wt/vol) agarose containing Tris-borate/EDTA buffer in a PFGE apparatus (CHEF DR III; Bio-Rad Laboratories), according to the following protocol: block I: 9 hours, 120° included angle, 5.5 V/cm, 30 to 18-s switch; block II: 6 hours, 117° included angle, 4.5 V/cm, 18 to 9-s switch; block III: 6 hours, 112° included angle, 4.0 V/cm, 9 to 5-s switch. The gel was then stained with SYBR Gold (1 part in 10,000 in water; Invitrogen) and analyzed by the AlphaImager system (ProteinSimple). Relative DSB levels were assessed by comparing DSB signals for each treatment to the background levels observed in untreated conditions using Image J. Data were analyzed with GraphPad Prism software.

### Microfluidic-assisted replication track analysis

maRTA was performed as previously described with some modifications [31]. Briefly, 36 hours after siRNA transfection, HeLa cells were pulse-labeled with 50 μM iododeoxyuridine (IdU) for 40 min. Cells were then treated or not with 2 mM HU for 5 hours or 16 hours. The cells were released in fresh medium containing 50 μM of chlorodeoxyuridine (CldU) for 40 min. Cells were then harvested and embedded into agarose plugs containing 20,000 cells/plug. After proteinase K digestion and agarose digestion by beta-agarase, DNA fibers were stretched on 3-aminopropyltriethoxysilane coated slides (LabScientific) using polydimethylsiloxane molds fashioned with micro-capillary channels prepared as described [31]. DNA fibers were then denatured in 2.5 M HCl, and probed with the following antibodies: mouse IgG_1_ anti-BrdU/IdU (clone BD44, Becton Dickinson), rat anti-BrdU/CldU (clone B1/75, Bio-Rad OBT0030), and mouse IgG_2a_ anti-ssDNA (clone 16–19, Millipore). Secondary antibodies included Alexa Fluor 488 anti-mouse IgG_1_, Alexa Fluor 594 anti-rat, and Alexa Fluor 647 anti-mouse IgG_2a_, respectively (Life Technologies). Images were acquired on Leica DMI6000 epifluorescence microscope using Leica LAS-AF software. Signals were measured using NIH ImageJ software with custom-made modifications and the data analyzed with GraphPad Prism software.

### Statistics

Statistical evaluations of experiments with continuous variables (e.g., quantitation of SUMO-BLM and DSBs) or discrete variables in which the data was normal were carried out by paired Students t-test. Evaluations of experiments with non-normal discrete variables (e.g., focal counts) were analyzed by Mann-Whitney test. All p-values were two-sided.

## Supporting information

S1 Fig (A) 80% reduction of the levels of NSMCE2 after depletion with siRNA in HeLa cells as measured by Western blot and qPCR analysis. (B-D) Analysis of the construction of NSMCE2 null cells in the HEK293T cell line. (B) (Upper panel) Western blot analysis of normal, heterozygous, and two cell clones (clone 9 and clone 15) that are null for NSMCE2. HSC70 was used as a loading control. The homozygous null cell clones were both derived from the heterozygous mutant of *NSCME2* that carried a 10-bp deletion in exon 2 of *NSMCE2*. (Lower panel) Analysis by PCR and sequencing showed that clone 9 and clone 15 each contain the 10-bp deletion and a second clone-specific frameshift mutation. The sequence of the PAM site is denoted in blue and the sequence of the guide RNA is denoted in red. (C) Analysis by hemocytometer-based cell counting of cell proliferation of normal, heterozygous, and null cell clones of NSMCE2. The cell counting experiments indicated that the rate of proliferation of *NSMCE2+/+* cells is approximately 20 hours per division and of *NSMCE2-/-* cells 40 hours per division. (D) Flow cytometric analysis of the cell cycle. Cells were treated or not with 2 mM HU and then released (Rel) into normal medium for 6 hours. Cells were pulsed with 10 μM EdU prior to harvest and processing for flow cytometry. The NSCME2 null cells exhibit a mild G1 delay. Normal cells were pulsed with EdU for 20 min and NSMCE2 null cells were pulsed for 40 min to account for the slower cell cycle.
(TIF)

S2 Fig (A) Representative Western blots of HeLa cells transfected with control or two different siRNAs against NSMCE2 and treated or not with 2 mM HU for 24 hours. Multiple loading controls (HSP90) are shown for separate gel runs and Westerns of the same cell lysate. (B) Western blot analysis of SMC5. For SMC5 experiments, β-actin was used as a loading control.
(TIF)

S3 Fig (A) Complementation of accumulation of BLM foci by transfection of siRNA-resistant NSMCE2 cDNA construct. HeLa cells were exposed to control or NSMCE2 siRNAs and were treated with 2 mM HU for 24 hours. Box and whisker plots represent distributions of the number of BLM foci per cell. The median values are shown in boxes. At least 10,000 BLM foci were analyzed in each experimental condition. Three independent experiments were performed. (B) A representative image of the colocalization of RPA (red) and RAD51 (green) in HeLa cells exposed to 2 mM HU for 24 hours prior to fixation (upper panel). Quantitation of the area of RAD51 foci (lower panel). Mean and standard error are shown. At least 10,000 RAD51 foci were analyzed in each experimental condition. Three independent experiments were performed. (C) Colocalization of RAD51 and EdU in HU-treated cells. Representative images of control and NSMCE2-depleted HeLa cells exposed to 2 mM HU for 24 hours. EdU was incorporated for 12 min prior to HU treatment. After HU, cells were fixed and stained with RAD51. Images show the merge of EdU (green) and RAD51 (red) channels. (D) Reduced accumulation of RPA foci in HU-treated, NSMCE2-deficient U2OS cells. Box and whiskers plot represent distributions of the number of RPA foci in cells exposed to control or NSMCE2 siRNA and treated or not with 2 mM HU for 24 hours. The median values are shown in boxes. Three independent experiments were performed. (E) Complementation of accumulation of RPA foci by transfection of siRNA-resistant NSMCE2 cDNA construct. HeLa cells were exposed to control or NSMCE2 siRNAs and treated with 2 mM HU for 24 hours. Box and whiskers plot represent the distributions of the number of RPA foci per cell. The median values are shown in boxes. Three independent experiments were performed. (F) Reduced accumulation of chromatin-bound RPA in HU-treated NSMCE2 null cells compared to HU-treated normal HEK293T cells. Western blot analysis of levels of chromatin-bound RPA (RPA p70 subunit). Cells were treated or not with 2 mM HU for 16 hours. The M fraction contains equal parts of the cytoplasmic and nucleoplasmic fractions. The C fraction contains the chromatin-bound material. The red carets point to the HU-induced chromatin-bound RPA. Four independent experiments were performed. (G) Quantitation of the experiment shown in F. (H) Reduced levels of ssDNA in HU-treated NSMCE2-deficient cells. Quantitation of immunofluorescence analysis of BrdU to measure exposed ssDNA in non-denaturing conditions (left panel). HeLa cells were exposed to control or NSMCE2 siRNAs and treated or not with 2 mM HU for 24 hours. The bar represents median values of the numbers of BrdU foci and the error bar represent the SEM values from three independent experiments. Representative images of BrdU foci are shown (right panel). (I) Similar levels of SCEs in normal HEK293T cells and NSMCE2 null cells. Box and whiskers plots represent the numbers of SCEs per metaphase. A minimum of 14 metaphases were scored in two independent experiments. (J) Reduced levels of γ-H2AX in HU-treated, NSMCE2-deficient cells. Flow cytometric analysis of γ-H2AX response in HeLa cells. Mean and standard deviation is shown. To the right of the bar graph are representative histograms showing γ-H2AX induction. Shaded histograms represent the treated cell populations. Three independent experiments were performed. (K) Complementation of accumulation of γ-H2AX foci by transfection of siRNA-resistant NSMCE2 cDNA construct. Quantitative analysis of γ-H2AX foci (upper panel). Box and whisker plots represent distributions of the number of γ-H2AX foci per cell. The median values are shown in boxes. At least 10,000 γ-H2AX foci were analyzed in each experimental condition. Below the bar graph are representative immunofluorescence images. Three independent experiments were performed.
(TIF)

S4 Fig Low magnification images of cells analyzed for the indirect immunofluorescence experiments. (A) BLM is retained in PML nuclear bodies in NSMCE2-deficient cells and the numbers of BLM foci induced by HU are reduced in NSMCE2-deficient cells. Merged images of untreated or HU-treated, control- and NSMCE2-depleted HeLa cells stained with antibodies to BLM (red) and PML (green) and counter stained with DAPI. Depletion of NSMCE2 is associated with increased numbers of PML nuclear bodies. In HU-treated, control-depleted cells, BLM moves to stalled replication forks. In HU-treated NSMCE2-depleted cells, BLM remains associated with PML. (B) Reduced accumulation of RPA foci in HU-treated, NSMCE2-deficient cells. Images of HeLa cells stained with phalloidin, DAPI, anti-RPA p32 antibodies. Actin staining also revealed striking morphological changes in NSMCE2-deficient HeLa cells. (C) Over-accumulation of RAD51 foci in HU-treated, NSMCE2-deficient cells. Images of HeLa cells stained with DAPI and anti-RAD51 antibodies. (D) Reduced accumulation of BrdU foci in HU-treated, NSMCE2-deficient cells. Images are of HeLa cells stained with DAPI and anti-BrdU antibodies. Cells were incubated with 10 μM BrdU for 48 hours, treated with HU for 24 hours, then processed for immunofluorescence. (E) Excess accumulation of 53BP1 foci in HU-treated NSMCE2-deficient cells. Images of HeLa cells treated with HU for 24 hours then released for 24 hours and stained with DAPI and anti-53BP1 antibodies.
(TIF)

S5 Fig (A) DSBs that accumulate after release from HU block are replication-dependent. HeLa cells were treated or not with 2 mM HU or 10 μM aphidicolin for 24 hours. The HU-treated cells were released into normal medium or medium that contained 10 μM aphidicolin for an additional 24 hours. The cells were analyzed by PFGE. Means and SD are shown. Three independent experiments were performed. (B) Similar cell-cycle distributions in HeLa cells transfected with control or NSMCE2 siRNA after release from HU block. Flow cytometric analysis of the cell cycle after HU treatment and release. 24 hours after transfection, cells were treated or not with 2 mM HU for 24 hours prior to release into normal medium for 40 min, 6 hours, or 12 hours. The cells were then pulsed with 20 μM EdU for 20 min prior to harvest and staining with click reagents. A minimum of 10,000 events were recorded in each experimental condition. (C) No induction of DSBs in NSMCE2 null cells treated with HU. Quantitation of PFGE analysis of HEK293T cells treated or not with 2 mM HU for 24 hours prior to release into normal medium for 12 hours (upper panel). The bar graph shows the mean fold change values, normalized to the untreated normal HEK293T mean, and SEM values from three independent experiments. A representative gel image of one experiment is shown below the graph (lower panel). (D) Similar levels of apoptosis in HeLa cells exposed to control and NSMCE2 siRNAs after treatment with 2 mM HU for 24 hours and release into normal medium for 24 hours. Analysis of apoptosis staining with propidium iodide and antibodies against AnnexinV. A minimum of 10,000 events were recorded in each experimental condition. (E) Reduced levels of γ-H2AX in HU-treated NSMCE2 null cells. Graph showing fold change in median levels of HU-induced γ-H2AX in normal HEK293T cells, the heterozygous NSMCE2+/- cells, and the two NSMCE2-/- clones 9 and 15. Data were normalized to HU-treated normal HEK293T cells. Two independent experiments were performed.
(TIF)

S6 Fig (A) Persistence of RAD51 at collapsed replication forks in NSMCE2 null cells. Immunofluorescence analysis of HEK293T cells treated or not 2 mM HU for 24 hours prior to release for 2 hours, 4 hours, or 6 hours before staining, imaging, and quantitation. Each point on the graph represents the median value and the error bar represent SEM values from randomly binned averages of 10 cells from at least 50 cells in each experimental condition. Two independent experiments were performed. (B) Representative images of data shown in (A) showing co-localization of γ-H2AX, RPA, and RAD51 in HEK293T cells after 24 hours treatment with HU and release into normal medium for 2 hours.
(TIF)

S7 Fig Mitotic damage in HU-treated NSMCE2-deficient cells. (A) Quantitative analysis of G1 arrest after release of control and NSMCE2-depleted HeLa cells from HU block into normal medium for 24 hours. (B) Quantitative analysis of abnormal anaphases encountered after release from HU block (left panel). HeLa cells were exposed to control or NSMCE2 siRNAs and treated or not with 2 mM HU for 24 hours, then released into fresh media for 16 hours before fixation and staining. The graph plots percent values of normal mitoses (blue), mitoses with anaphase bridges (red), and mitoses with lagging chromosomes (green). The value shown above the graph is the percent of abnormal mitosis scored. Representative images of normal mitosis (B’), mitosis with an anaphase bridge (red caret in B”), and mitosis with a lagging chromosome (red caret in B”‘) are shown (right panel). At least 100 anaphase cells were analyzed in each experimental condition. Three independent experiments were performed. (C) Quantitative analysis of micronuclei formation after release from HU block (left panel). Experiment was performed three times and analyzed by chi square test. A representative image is shown to the right of the graph. Scale bars represent 10 microns. (D) Quantitation of 53BP1 nuclear bodies/cell using bins of 1, 2, and 3 or more bodies/cell. HeLa cells were exposed to control or NSMCE2 siRNAs and treated with 2 mM HU for 24 hours, then released into fresh media for 24 hours before fixation and staining. Each bar represents the mean percent of total cells observed in each class and the error bars represent the SEM values. Flow cytometric analysis showed that the majority of cells are in G1 (see panel A). Cells with large nuclei indicative of being in the G2 phase were excluded from the quantitation. Three independent experiments were performed. (E) Quantitative analysis of area of the nucleus inhabited by 53BP1 signal after release from HU block based on the same data in panel D. Box and whisker plot represents area distribution per cell in at least 250 cells in each experimental condition. (F) Representative images of cells stained for 53BP1 analyzed in panels D and E. (G) Flow cytometric analysis of the effect of the treatment schedule to enrich for mitotic cells used in the experiments shown in Fig 3. HeLa cells were transfected with control or NSMCE2 siRNAs. After 24 hours, they were treated or not with 2 mM HU for 24 hours followed by release into medium containing 7.5 μM RO-3306 for 15 hours, followed by release into normal media for 1 hour. Cells were harvested, fixed, and stained with 7-AAD. 10,000 events were analyzed in each experimental condition. This experiment was performed two times.
(TIF)

S8 Fig Increased γ-H2AX signal in metaphase chromosomes in HU-treated NSMCE2 null cells. (A) Quantitation of median fluorescence intensity of γ-H2AX on phospho-histone H3-positive chromosomes. Cells were treated with 2 mM HU for 24 hours, released into medium containing 7.5 μM RO-3306 for 10 hours (normal HEK293T) or 20 hours (NSMCE2 null) to block cells at the G2/M boundary, and then released into normal medium and harvested at the indicated times for analysis of metaphase chromosomes. Regions of interest were drawn using Image J based on the DAPI signal in phospho-histone H3-positive (serine 10) cells and γ-H2AX signal was quantified. Two independent experiments were performed. (B) Representative images of metaphase chromosomes stained with γ-H2AX, anti-phospho-histone H3 and DAPI.
(TIF)

S9 Fig Increased chromosome aberrations in untreated and HU-treated NSMCE2 null cells. (A) Representative images of metaphases prepared from normal HEK293T and NSMCE null cells. (B) Quantitation of chromosome aberrations identified in untreated and HU-treated normal HEK293T and NSMCE2 null cells. Representative chromosome images are shown below the grid of counts of chromosome aberrations. Total indicates the number of chromosomes scored. 1, chromatid break. 2, chromosome break. 3, chromosome gap. 4, telomere fusion. 5, tri-radial. 6, quadriradial. 7, secondary constriction. Approximately 25 metaphases were analyzed from each of two experiments.
(TIF)

S10 Fig *RAD51* and *NSMCE2* are epistatic with respect to HU-induced phenotypes. Analysis of HU-induced phenotypes in normal HEK293T and NSMCE2 null cells in which RAD51 levels were reduced or not by siRNA-mediated depletion. (A) Analysis by flow cytometry of the fluorescence intensity of γ-H2AX after treatment with HU for 24 hours followed by release into normal medium for 6 hours (Rel). The error bars represent the SD of median fluorescence intensity from a minimum of 10,000 events in five independent experiments. (B) Analysis by PFGE of DSBs after treatment with HU for 24 hours followed by release into medium for 6 hours (Rel). The bar graph represents the mean fold change in DSBs detected by PFGE normalized to untreated normal HEK293T cells exposed to control siRNA. The error bars represent the SD of three independent experiments. The inset gel shows the results from one experiment. (C) A representative histogram of the median fluorescence intensity of γ-H2AX from one of the experiments shown in A. (D) Western analysis of RAD51 levels from samples prepared for one of the PFGE experiments shown in (B).
(TIF)

S1 Data The underlying numerical data for each figure in the article is tabulated in each separate tab of the excel file. The tabs are labeled F1A for Fig 1A, F1C for Fig 1C, and so on. Image files are not included. The tabulated data in each cell is described in the legend in each tab. The abbreviations for column and row headings follow the main text. See figure legends for more details.
(XLSX)
