# Supplementary figures and images for: Rescue of collapsed replication forks is dependent on NSMCE2 to prevent mitotic DNA damage
Source: PLoS Genet. 2019 Feb 8;15(2):e1007942. doi: 10.1371/journal.pgen.1007942 (PMC6383951; doi:10.1371/journal.pgen.1007942)

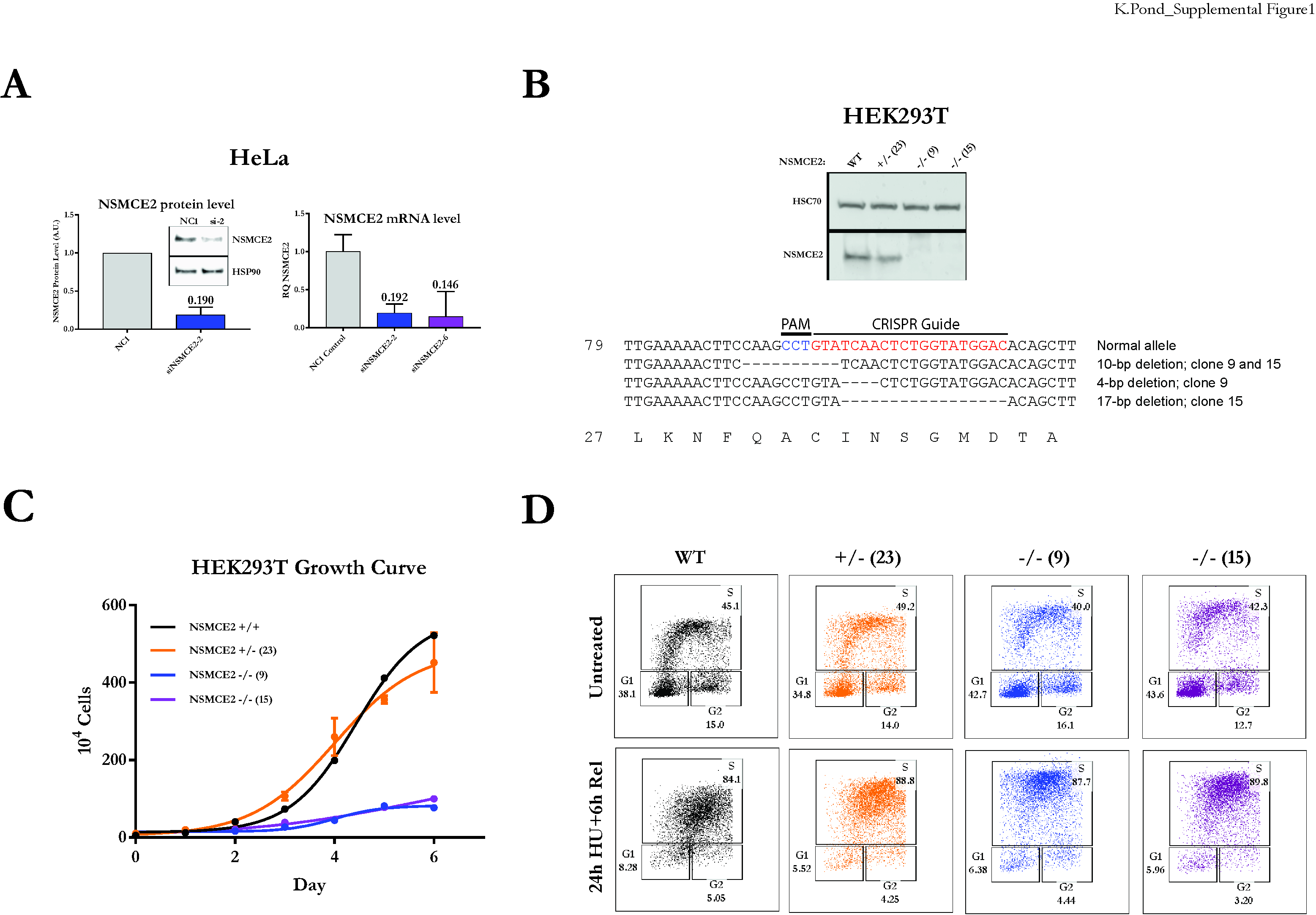

Supplement: S1 Fig — (A) 80% reduction of the levels of NSMCE2 after depletion with siRNA in HeLa cells as measured by Western blot and qPCR analysis. (B-D) Analysis of the construction of NSMCE2 null cells in the HEK293T cell line. (B) (Upper panel) Western blot analysis of normal, heterozygous, and two cell clones (clone 9 and clone 15) that are null for NSMCE2. HSC70 was used as a loading control. The homozygous null cell clones were both derived from the heterozygous mutant of NSCME2 that carried a 10-bp deletion in exon 2 of NSMCE2. (Lower panel) Analysis by PCR and sequencing showed that clone 9 and clone 15 each contain the 10-bp deletion and a second clone-specific frameshift mutation. The sequence of the PAM site is denoted in blue and the sequence of the guide RNA is denoted in red. (C) Analysis by hemocytometer-based cell counting of cell proliferation of normal, heterozygous, and null cell clones of NSMCE2. The cell counting experiments indicated that the rate of proliferation of NSMCE2+/+ cells is approximately 20 hours per division and of NSMCE2-/- cells 40 hours per division. (D) Flow cytometric analysis of the cell cycle. Cells were treated or not with 2 mM HU and then released (Rel) into normal medium for 6 hours. Cells were pulsed with 10 μM EdU prior to harvest and processing for flow cytometry. The NSCME2 null cells exhibit a mild G1 delay. Normal cells were pulsed with EdU for 20 min and NSMCE2 null cells were pulsed for 40 min to account for the slower cell cycle. (TIF) [file pgen.1007942.s001.tif]

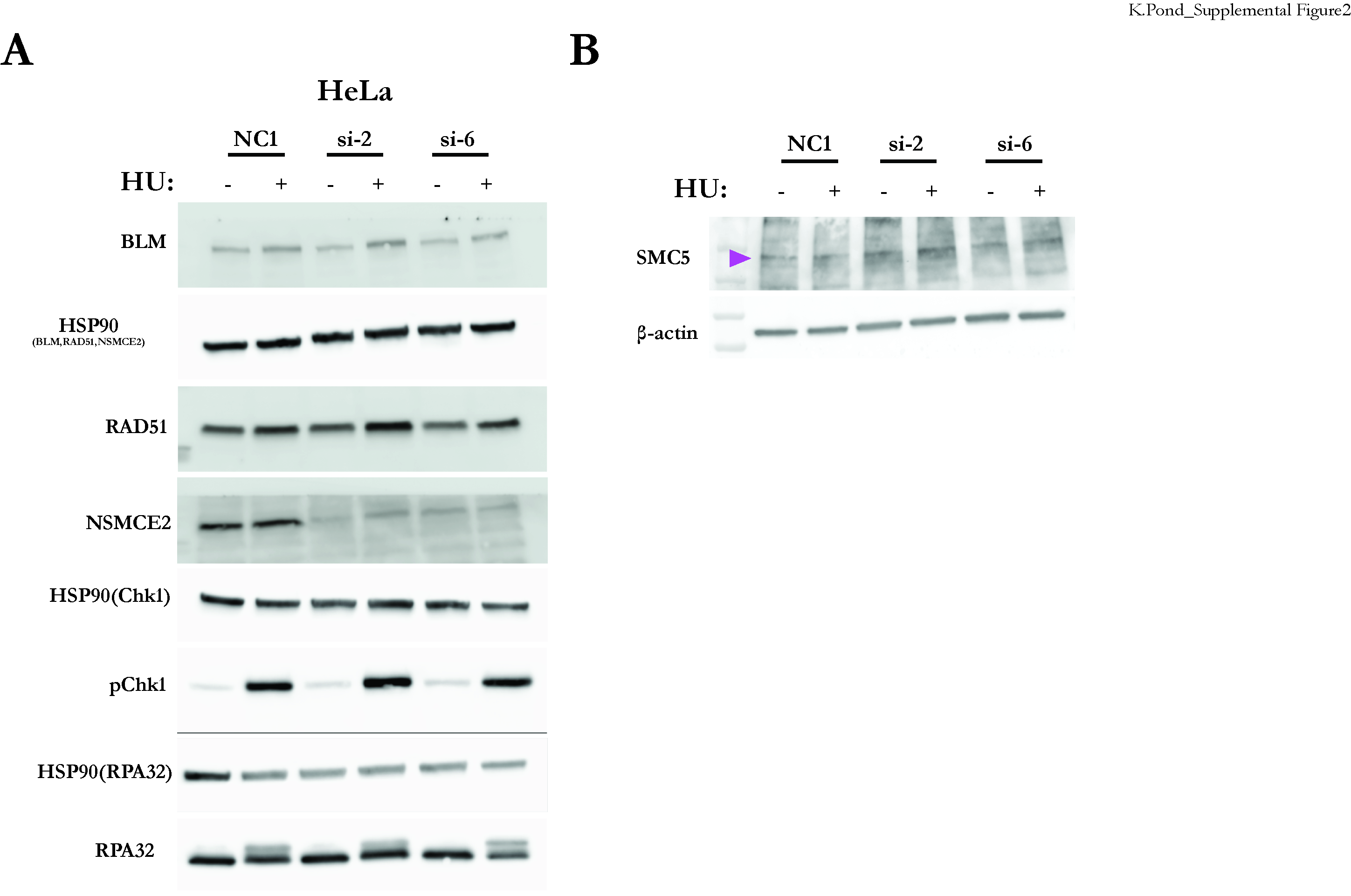

Supplement: S2 Fig — (A) Representative Western blots of HeLa cells transfected with control or two different siRNAs against NSMCE2 and treated or not with 2 mM HU for 24 hours. Multiple loading controls (HSP90) are shown for separate gel runs and Westerns of the same cell lysate. (B) Western blot analysis of SMC5. For SMC5 experiments, β-actin was used as a loading control. (TIF) [file pgen.1007942.s002.tif]

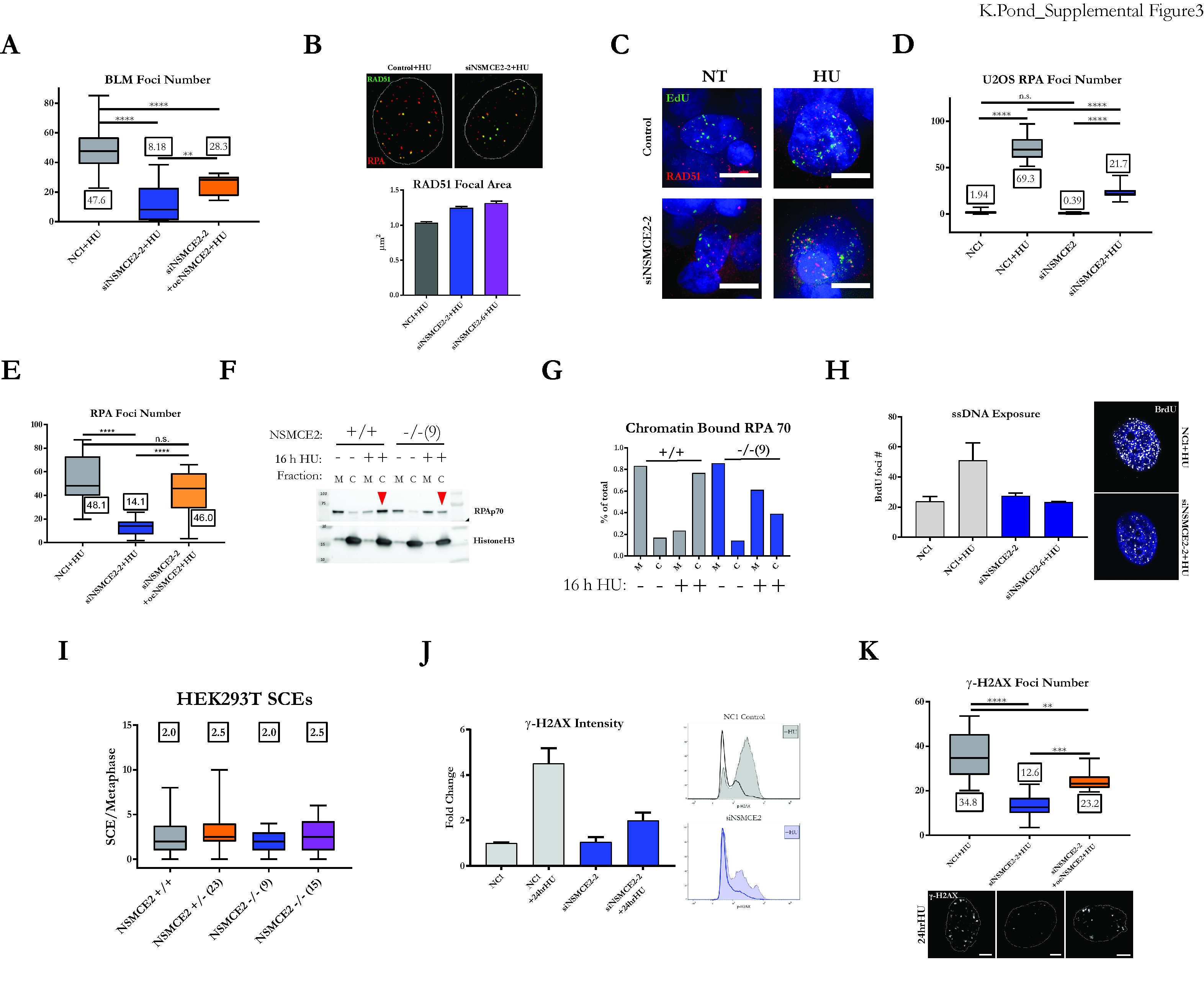

Supplement: S3 Fig — (A) Complementation of accumulation of BLM foci by transfection of siRNA-resistant NSMCE2 cDNA construct. HeLa cells were exposed to control or NSMCE2 siRNAs and were treated with 2 mM HU for 24 hours. Box and whisker plots represent distributions of the number of BLM foci per cell. The median values are shown in boxes. At least 10,000 BLM foci were analyzed in each experimental condition. Three independent experiments were performed. (B) A representative image of the colocalization of RPA (red) and RAD51 (green) in HeLa cells exposed to 2 mM HU for 24 hours prior to fixation (upper panel). Quantitation of the area of RAD51 foci (lower panel). Mean and standard error are shown. At least 10,000 RAD51 foci were analyzed in each experimental condition. Three independent experiments were performed. (C) Colocalization of RAD51 and EdU in HU-treated cells. Representative images of control and NSMCE2-depleted HeLa cells exposed to 2 mM HU for 24 hours. EdU was incorporated for 12 min prior to HU treatment. After HU, cells were fixed and stained with RAD51. Images show the merge of EdU (green) and RAD51 (red) channels. (D) Reduced accumulation of RPA foci in HU-treated, NSMCE2-deficient U2OS cells. Box and whiskers plot represent distributions of the number of RPA foci in cells exposed to control or NSMCE2 siRNA and treated or not with 2 mM HU for 24 hours. The median values are shown in boxes. Three independent experiments were performed. (E) Complementation of accumulation of RPA foci by transfection of siRNA-resistant NSMCE2 cDNA construct. HeLa cells were exposed to control or NSMCE2 siRNAs and treated with 2 mM HU for 24 hours. Box and whiskers plot represent the distributions of the number of RPA foci per cell. The median values are shown in boxes. Three independent experiments were performed. (F) Reduced accumulation of chromatin-bound RPA in HU-treated NSMCE2 null cells compared to HU-treated normal HEK293T cells. Western blot analysis of levels of chromatin-bound R [file pgen.1007942.s003.tif]

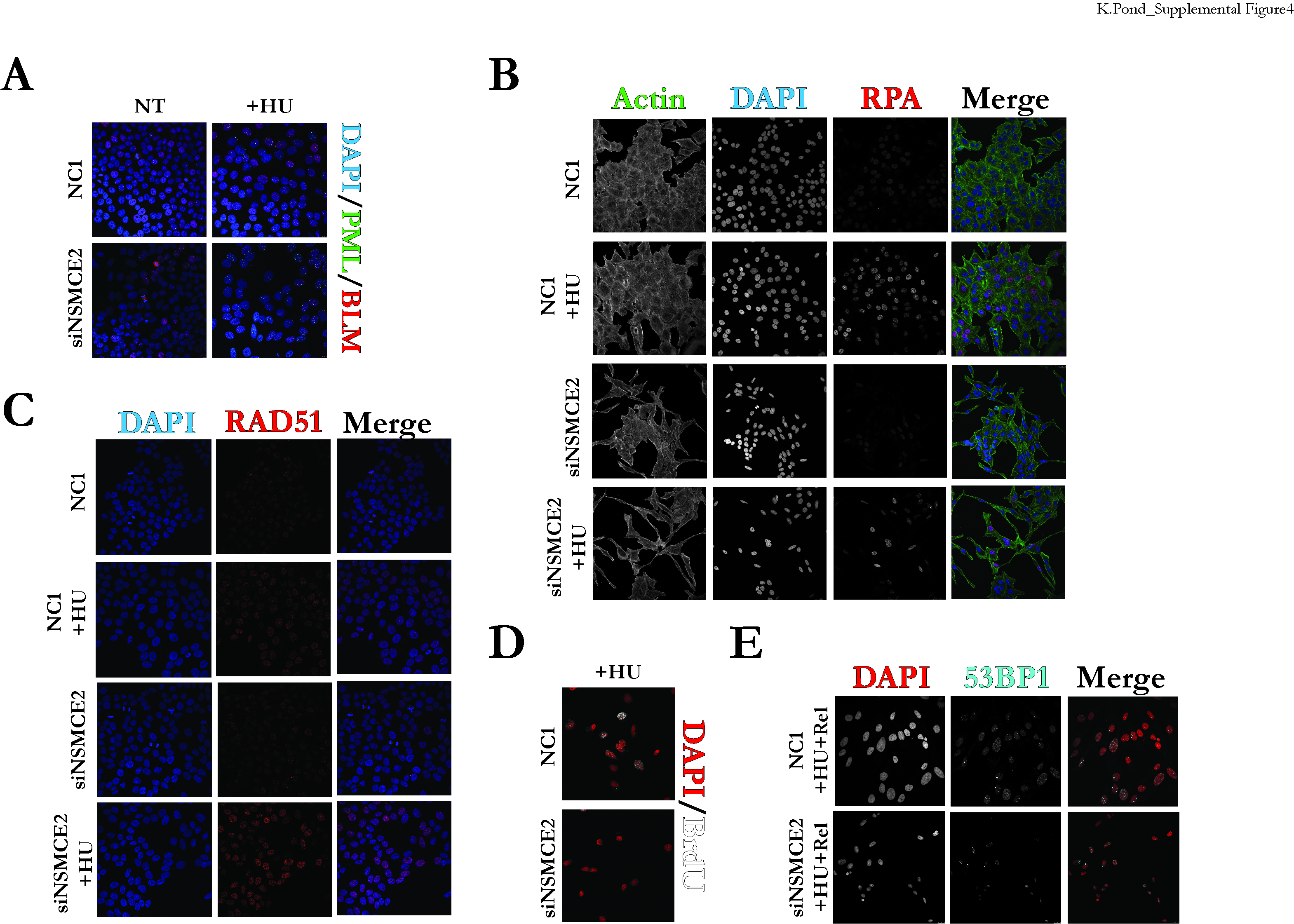

Supplement: S4 Fig — (A) BLM is retained in PML nuclear bodies in NSMCE2-deficient cells and the numbers of BLM foci induced by HU are reduced in NSMCE2-deficient cells. Merged images of untreated or HU-treated, control- and NSMCE2-depleted HeLa cells stained with antibodies to BLM (red) and PML (green) and counter stained with DAPI. Depletion of NSMCE2 is associated with increased numbers of PML nuclear bodies. In HU-treated, control-depleted cells, BLM moves to stalled replication forks. In HU-treated NSMCE2-depleted cells, BLM remains associated with PML. (B) Reduced accumulation of RPA foci in HU-treated, NSMCE2-deficient cells. Images of HeLa cells stained with phalloidin, DAPI, anti-RPA p32 antibodies. Actin staining also revealed striking morphological changes in NSMCE2-deficient HeLa cells. (C) Over-accumulation of RAD51 foci in HU-treated, NSMCE2-deficient cells. Images of HeLa cells stained with DAPI and anti-RAD51 antibodies. (D) Reduced accumulation of BrdU foci in HU-treated, NSMCE2-deficient cells. Images are of HeLa cells stained with DAPI and anti-BrdU antibodies. Cells were incubated with 10 μM BrdU for 48 hours, treated with HU for 24 hours, then processed for immunofluorescence. (E) Excess accumulation of 53BP1 foci in HU-treated NSMCE2-deficient cells. Images of HeLa cells treated with HU for 24 hours then released for 24 hours and stained with DAPI and anti-53BP1 antibodies. (TIF) [file pgen.1007942.s004.tif]

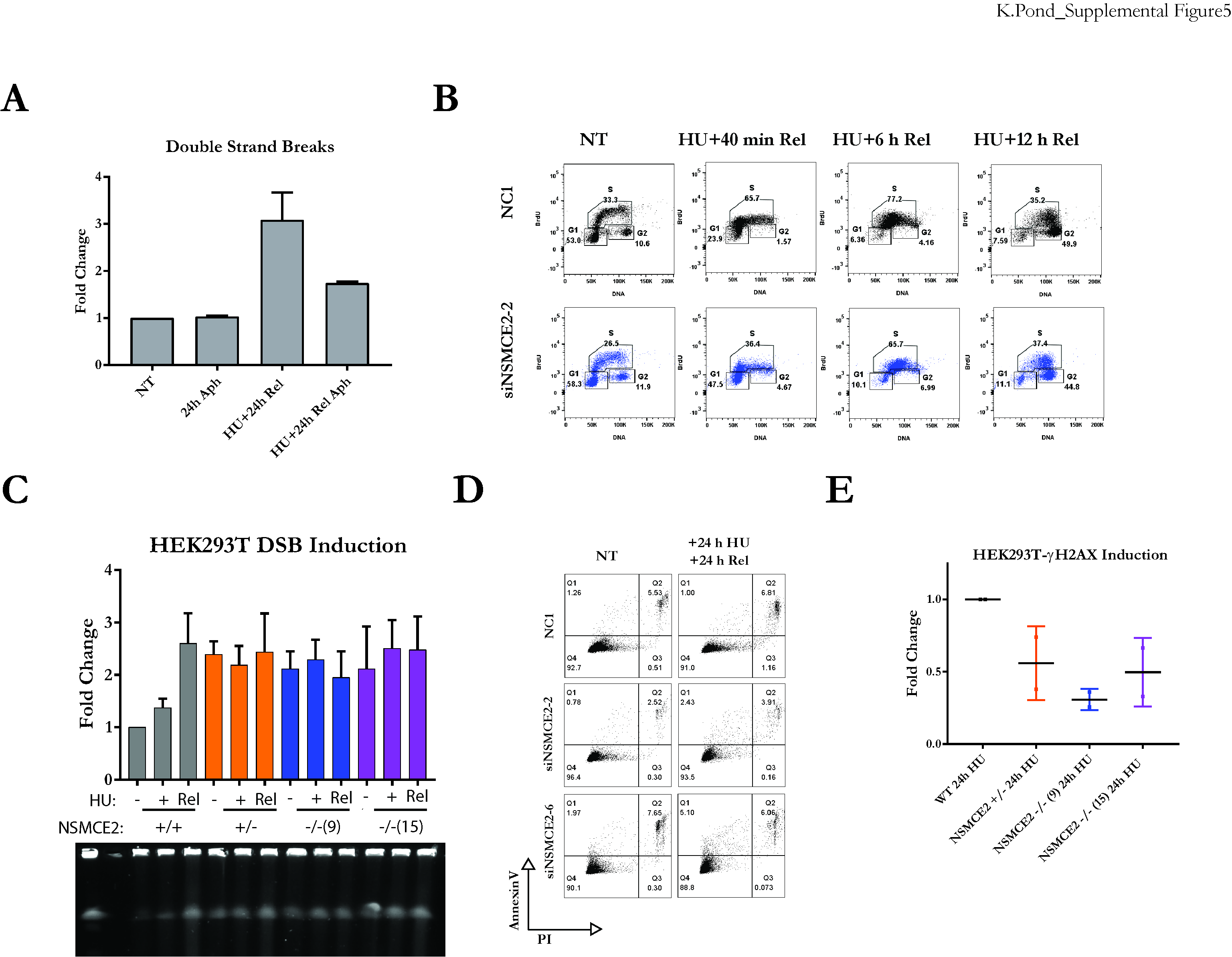

Supplement: S5 Fig — (A) DSBs that accumulate after release from HU block are replication-dependent. HeLa cells were treated or not with 2 mM HU or 10 μM aphidicolin for 24 hours. The HU-treated cells were released into normal medium or medium that contained 10 μM aphidicolin for an additional 24 hours. The cells were analyzed by PFGE. Means and SD are shown. Three independent experiments were performed. (B) Similar cell-cycle distributions in HeLa cells transfected with control or NSMCE2 siRNA after release from HU block. Flow cytometric analysis of the cell cycle after HU treatment and release. 24 hours after transfection, cells were treated or not with 2 mM HU for 24 hours prior to release into normal medium for 40 min, 6 hours, or 12 hours. The cells were then pulsed with 20 μM EdU for 20 min prior to harvest and staining with click reagents. A minimum of 10,000 events were recorded in each experimental condition. (C) No induction of DSBs in NSMCE2 null cells treated with HU. Quantitation of PFGE analysis of HEK293T cells treated or not with 2 mM HU for 24 hours prior to release into normal medium for 12 hours (upper panel). The bar graph shows the mean fold change values, normalized to the untreated normal HEK293T mean, and SEM values from three independent experiments. A representative gel image of one experiment is shown below the graph (lower panel). (D) Similar levels of apoptosis in HeLa cells exposed to control and NSMCE2 siRNAs after treatment with 2 mM HU for 24 hours and release into normal medium for 24 hours. Analysis of apoptosis staining with propidium iodide and antibodies against AnnexinV. A minimum of 10,000 events were recorded in each experimental condition. (E) Reduced levels of γ-H2AX in HU-treated NSMCE2 null cells. Graph showing fold change in median levels of HU-induced γ-H2AX in normal HEK293T cells, the heterozygous NSMCE2+/- cells, and the two NSMCE2-/- clones 9 and 15. Data were normalized to HU-treated normal HEK293T cells. Two independent experiments we [file pgen.1007942.s005.tif]

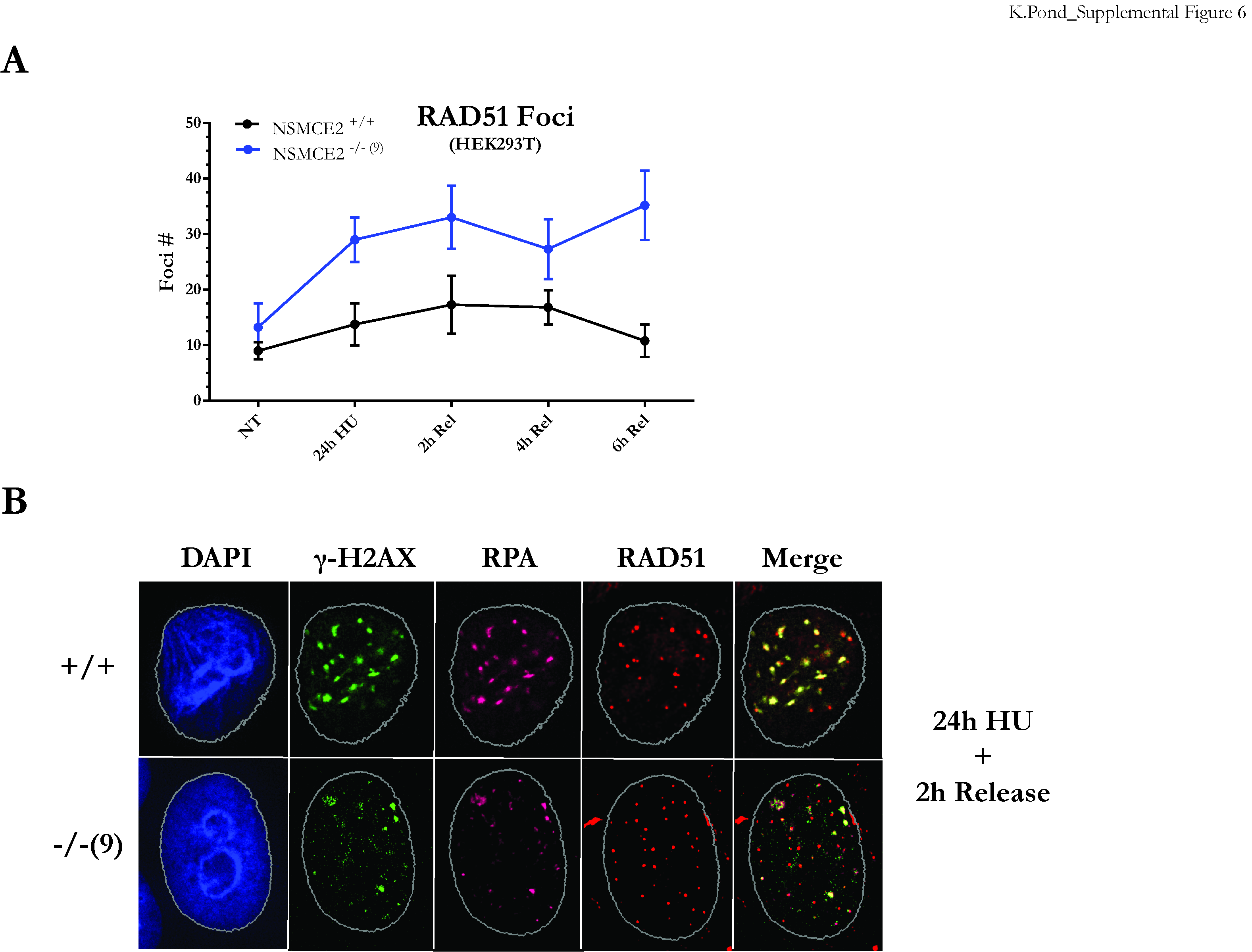

Supplement: S6 Fig — (A) Persistence of RAD51 at collapsed replication forks in NSMCE2 null cells. Immunofluorescence analysis of HEK293T cells treated or not 2 mM HU for 24 hours prior to release for 2 hours, 4 hours, or 6 hours before staining, imaging, and quantitation. Each point on the graph represents the median value and the error bar represent SEM values from randomly binned averages of 10 cells from at least 50 cells in each experimental condition. Two independent experiments were performed. (B) Representative images of data shown in (A) showing co-localization of γ-H2AX, RPA, and RAD51 in HEK293T cells after 24 hours treatment with HU and release into normal medium for 2 hours. (TIF) [file pgen.1007942.s006.tif]

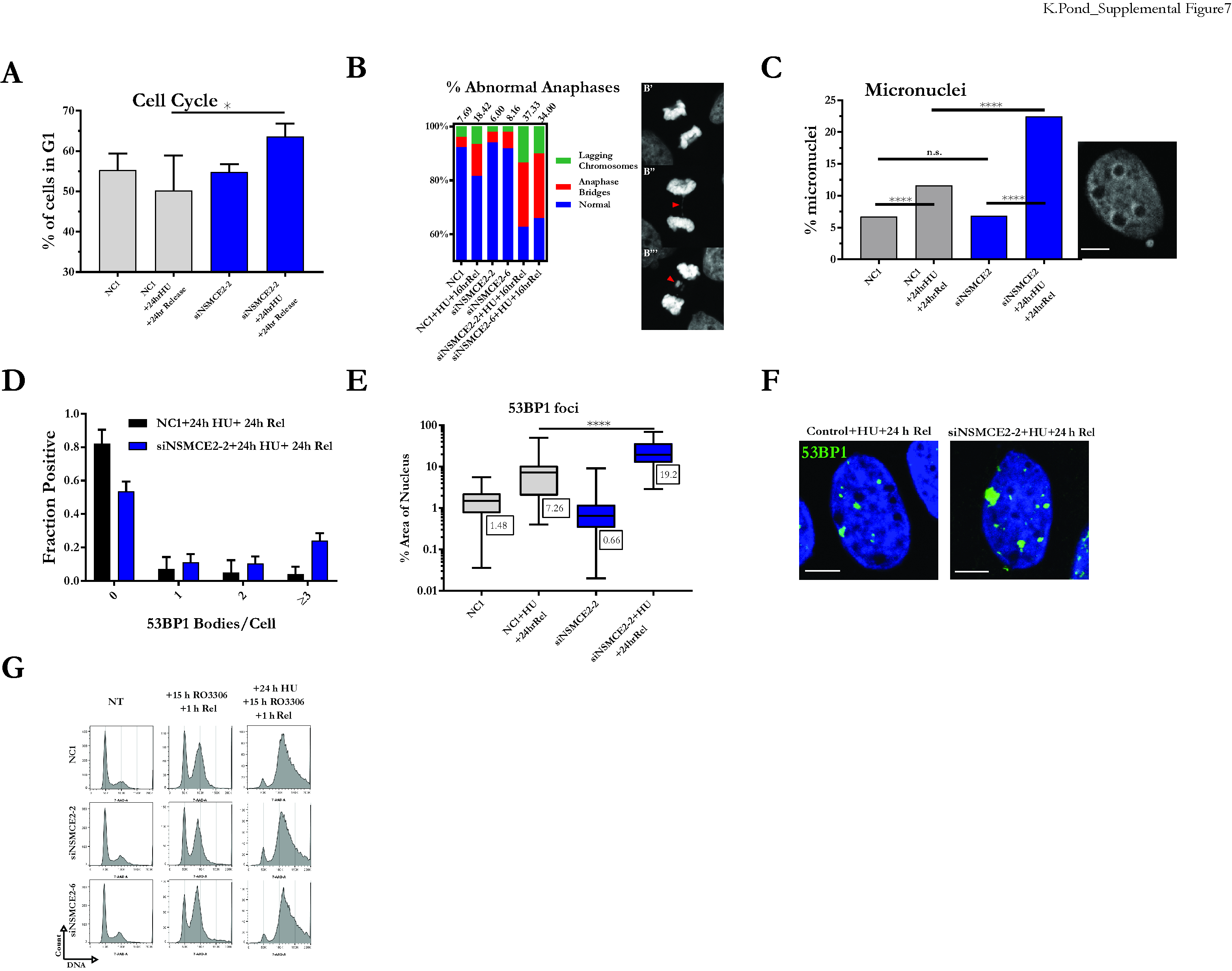

Supplement: S7 Fig — (A) Quantitative analysis of G1 arrest after release of control and NSMCE2-depleted HeLa cells from HU block into normal medium for 24 hours. (B) Quantitative analysis of abnormal anaphases encountered after release from HU block (left panel). HeLa cells were exposed to control or NSMCE2 siRNAs and treated or not with 2 mM HU for 24 hours, then released into fresh media for 16 hours before fixation and staining. The graph plots percent values of normal mitoses (blue), mitoses with anaphase bridges (red), and mitoses with lagging chromosomes (green). The value shown above the graph is the percent of abnormal mitosis scored. Representative images of normal mitosis (B’), mitosis with an anaphase bridge (red caret in B”), and mitosis with a lagging chromosome (red caret in B”‘) are shown (right panel). At least 100 anaphase cells were analyzed in each experimental condition. Three independent experiments were performed. (C) Quantitative analysis of micronuclei formation after release from HU block (left panel). Experiment was performed three times and analyzed by chi square test. A representative image is shown to the right of the graph. Scale bars represent 10 microns. (D) Quantitation of 53BP1 nuclear bodies/cell using bins of 1, 2, and 3 or more bodies/cell. HeLa cells were exposed to control or NSMCE2 siRNAs and treated with 2 mM HU for 24 hours, then released into fresh media for 24 hours before fixation and staining. Each bar represents the mean percent of total cells observed in each class and the error bars represent the SEM values. Flow cytometric analysis showed that the majority of cells are in G1 (see panel A). Cells with large nuclei indicative of being in the G2 phase were excluded from the quantitation. Three independent experiments were performed. (E) Quantitative analysis of area of the nucleus inhabited by 53BP1 signal after release from HU block based on the same data in panel D. Box and whisker plot represents area distribution per cell in at least 2 [file pgen.1007942.s007.tif]

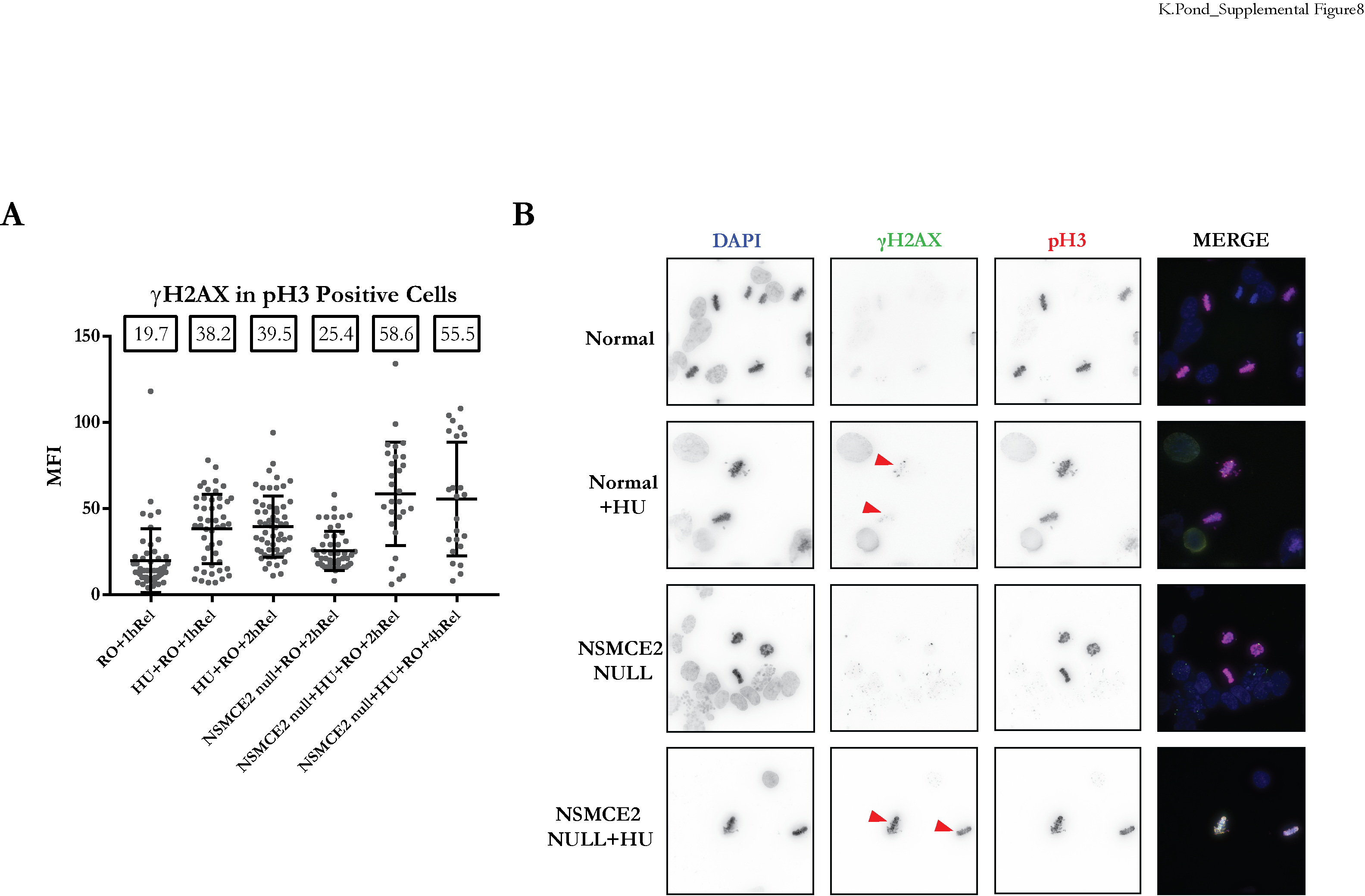

Supplement: S8 Fig — (A) Quantitation of median fluorescence intensity of γ-H2AX on phospho-histone H3-positive chromosomes. Cells were treated with 2 mM HU for 24 hours, released into medium containing 7.5 μM RO-3306 for 10 hours (normal HEK293T) or 20 hours (NSMCE2 null) to block cells at the G2/M boundary, and then released into normal medium and harvested at the indicated times for analysis of metaphase chromosomes. Regions of interest were drawn using Image J based on the DAPI signal in phospho-histone H3-positive (serine 10) cells and γ-H2AX signal was quantified. Two independent experiments were performed. (B) Representative images of metaphase chromosomes stained with γ-H2AX, anti-phospho-histone H3 and DAPI. (TIF) [file pgen.1007942.s008.tif]

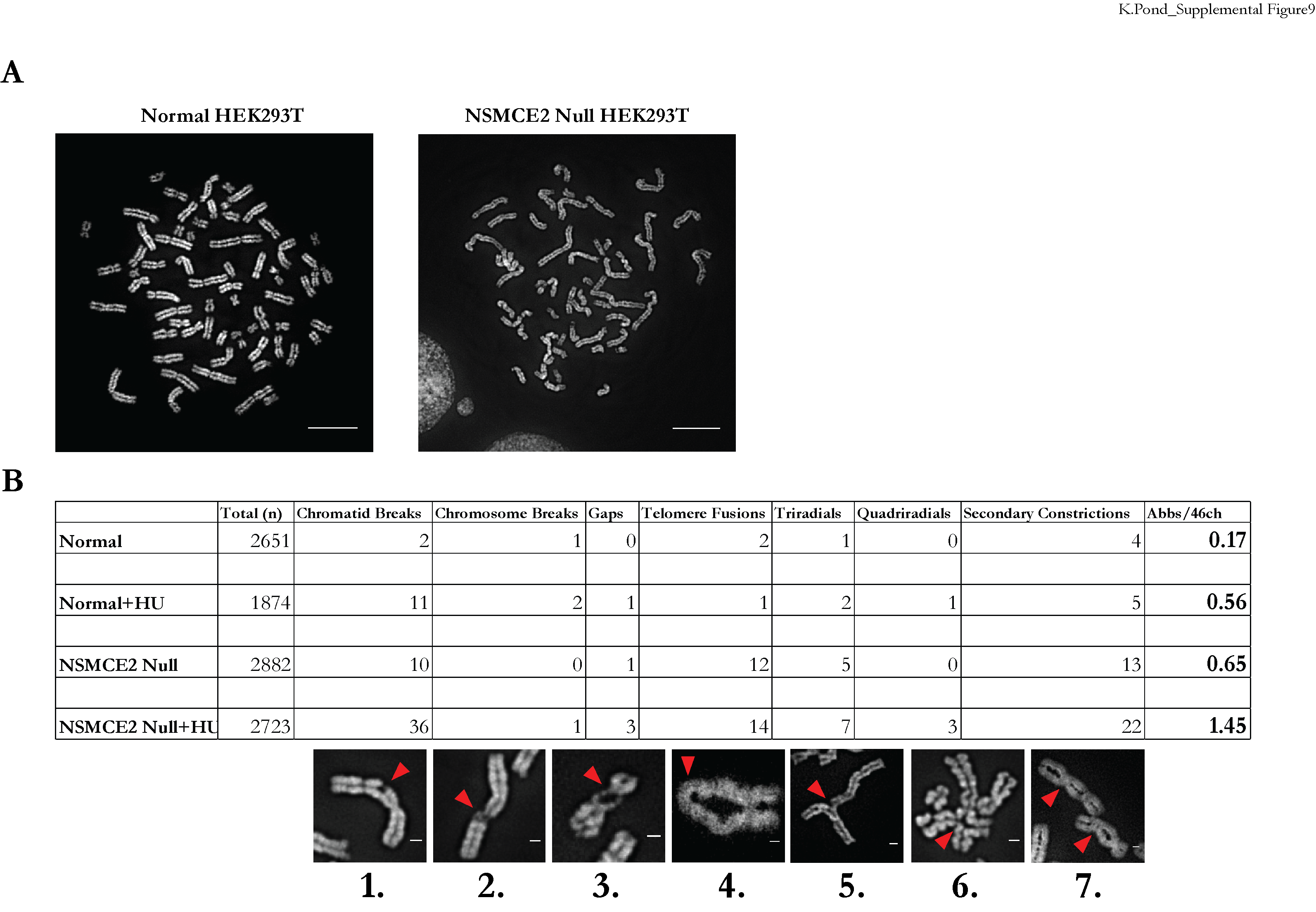

Supplement: S9 Fig — (A) Representative images of metaphases prepared from normal HEK293T and NSMCE null cells. (B) Quantitation of chromosome aberrations identified in untreated and HU-treated normal HEK293T and NSMCE2 null cells. Representative chromosome images are shown below the grid of counts of chromosome aberrations. Total indicates the number of chromosomes scored. 1, chromatid break. 2, chromosome break. 3, chromosome gap. 4, telomere fusion. 5, tri-radial. 6, quadriradial. 7, secondary constriction. Approximately 25 metaphases were analyzed from each of two experiments. (TIF) [file pgen.1007942.s009.tif]

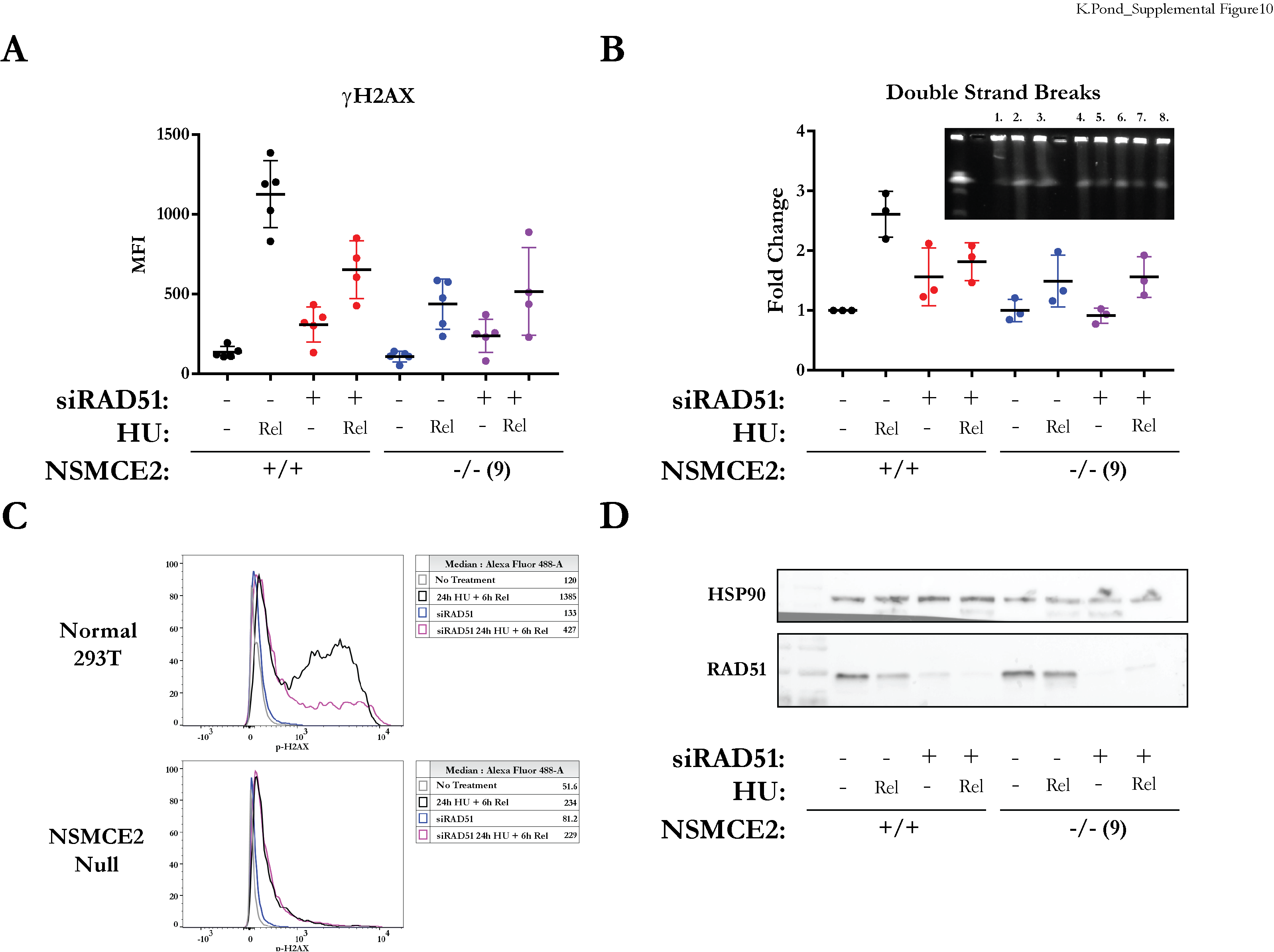

Supplement: S10 Fig — Analysis of HU-induced phenotypes in normal HEK293T and NSMCE2 null cells in which RAD51 levels were reduced or not by siRNA-mediated depletion. (A) Analysis by flow cytometry of the fluorescence intensity of γ-H2AX after treatment with HU for 24 hours followed by release into normal medium for 6 hours (Rel). The error bars represent the SD of median fluorescence intensity from a minimum of 10,000 events in five independent experiments. (B) Analysis by PFGE of DSBs after treatment with HU for 24 hours followed by release into medium for 6 hours (Rel). The bar graph represents the mean fold change in DSBs detected by PFGE normalized to untreated normal HEK293T cells exposed to control siRNA. The error bars represent the SD of three independent experiments. The inset gel shows the results from one experiment. (C) A representative histogram of the median fluorescence intensity of γ-H2AX from one of the experiments shown in A. (D) Western analysis of RAD51 levels from samples prepared for one of the PFGE experiments shown in (B). (TIF) [file pgen.1007942.s010.tif]
